# Fetal Alcohol Spectrum Disorders: An Overview from the Glia Perspective

**DOI:** 10.3389/fnint.2015.00065

**Published:** 2016-01-11

**Authors:** Clare J. Wilhelm, Marina Guizzetti

**Affiliations:** ^1^Research Service, VA Portland Health Care SystemPortland, OR, USA; ^2^Department of Psychiatry, Oregon Health and Science UniversityPortland, OR, USA; ^3^Department of Behavioral Neuroscience, Oregon Health and Science UniversityPortland, OR, USA

**Keywords:** astrocyte, microglia, oligodendrocyte, fetal alcohol syndrome, fetal alcohol spectrum disorders, neurodevelopment, animal models

## Abstract

Alcohol consumption during pregnancy can produce a variety of central nervous system (CNS) abnormalities in the offspring resulting in a broad spectrum of cognitive and behavioral impairments that constitute the most severe and long-lasting effects observed in fetal alcohol spectrum disorders (FASD). Alcohol-induced abnormalities in glial cells have been suspected of contributing to the adverse effects of alcohol on the developing brain for several years, although much research still needs to be done to causally link the effects of alcohol on specific brain structures and behavior to alterations in glial cell development and function. Damage to radial glia due to prenatal alcohol exposure may underlie observations of abnormal neuronal and glial migration in humans with Fetal Alcohol Syndrome (FAS), as well as primate and rodent models of FAS. A reduction in cell number and altered development has been reported for several glial cell types in animal models of FAS. *In utero* alcohol exposure can cause microencephaly when alcohol exposure occurs during the brain growth spurt a period characterized by rapid astrocyte proliferation and maturation; since astrocytes are the most abundant cells in the brain, microenchephaly may be caused by reduced astrocyte proliferation or survival, as observed in *in vitro* and *in vivo* studies. Delayed oligodendrocyte development and increased oligodendrocyte precursor apoptosis has also been reported in experimental models of FASD, which may be linked to altered myelination/white matter integrity found in FASD children. Children with FAS exhibit hypoplasia of the corpus callosum and anterior commissure, two areas requiring guidance from glial cells and proper maturation of oligodendrocytes. Finally, developmental alcohol exposure disrupts microglial function and induces microglial apoptosis; given the role of microglia in synaptic pruning during brain development, the effects of alcohol on microglia may be involved in the abnormal brain plasticity reported in FASD. The consequences of prenatal alcohol exposure on glial cells, including radial glia and other transient glial structures present in the developing brain, astrocytes, oligodendrocytes and their precursors, and microglia contributes to abnormal neuronal development, reduced neuron survival and disrupted brain architecture and connectivity. This review highlights the CNS structural abnormalities caused by *in utero* alcohol exposure and outlines which abnormalities are likely mediated by alcohol effects on glial cell development and function.

## Introduction

Prenatal alcohol exposure causes neurodevelopmental and physical alterations in the resultant offspring. The neurodevelopmental effects of *in utero* alcohol exposure are long-lasting affecting individuals throughout life and represent the major concerns associated with alcohol exposure during gestation (Guerri et al., 2009). Fetal Alcohol Syndrome (FAS) was the first characterized consequence of *in utero* alcohol exposure (Jones and Smith, 1973, 1975; Jones et al., 1973). Diagnosis of FAS requires the presence of three characteristics: growth retardation, distinct facial malformations (dysmorphia) and evidence of central nervous system (CNS) dysfunction. Both the Centers for Disease Control and Prevention (CDC) and the Institute of Medicine (IOM) have published guidelines for FAS diagnosis (Hoyme et al., 2005).

Alcohol-related neurodevelopmental disorder (ARND) is diagnosed when a maternal history of alcohol is established (Stratton et al., 1996). These individuals suffer from behavioral and cognitive impairments similar to those observed in FAS, yet lack the facial dysmorphia and/or growth retardation characteristic of FAS. For the diagnosis of ARND, structural, neurological or functional impairment in at least three of the followings domains must be present: achievement, adaptive behavior, attention, cognition, executive functioning, language, memory, motor skills, multisensory integration, or social communication (Warren et al., 2011).

More recently the term Fetal Alcohol Spectrum Disorders (FASD) has been introduced to describe the array of physical, behavioral, and learning impairments deriving from *in utero* alcohol exposure. Due to disruption of normal brain development, individuals with FASD may have a range of neurobehavioral deficits including impairments in attention, reaction time, visuospatial abilities, executive functions, motor skills, memory, language, and social and adaptive functions, and reduced IQ (Riley and McGee, 2005). As seen with other aspects of FASD, individuals afflicted with neurobehavioral deficits do not necessarily possess the characteristic facial features. However, those with prototypical FAS features generally exhibit more severe neurobehavioral deficits (Mattson and Riley, 1998). Approximately 25% of individuals with FAS fit the criteria for intellectual disability (an IQ lower than 70; Streissguth et al., 1997), making FAS the most common preventable cause of intellectual disability in the general population (Abel and Sokol, 1987). Given the range of cognitive impairments described above, it is not surprising that prenatal alcohol exposure is coincident with reduced academic performance and an increased frequency of learning disabilities (Howell et al., 2006). Indeed, attention deficit hyperactivity disorder (ADHD) is often diagnosed in FASD individuals with concordance rates ranging from 65–95% (Coles et al., 2002; Fryer et al., 2007; Rasmussen et al., 2010). Executive function is also impaired in individuals with FASD with deficits in response inhibition, concept formation, set shifting and planning (Guerri et al., 2009; Mattson et al., 2011). Individuals with FASD also exhibit weak grasp, poor hand/eye coordination, tremors, as well as gait and balance difficulties that can persist into adulthood and are indicative of motor control problems (Guerri et al., 2009; Mattson et al., 2011). Better therapeutics and biomarkers are needed to improve recognition and treatment for those suffering from FASD.

While FASD is not a diagnostic term, it should be noted that the American Psychiatric Association introduced for the first time into the appendix of the Diagnostic and Statistical Manual of Mental Disorders 5 (DSM-5) the new category called Neurobehavioral Disorder associated with Prenatal Alcohol Exposure (ND-PAE). The diagnosis requires evidence of prenatal alcohol exposure and CNS impairment specifically in three areas: cognition, self-regulation, and adaptive functioning.

With increased recognition, the estimated combined prevalence of FAS and partial FAS (pFAS) individuals with confirmed prenatal alcohol exposure, substantial CNS anomalies and most, though not all of the growth impairments and facial abnormalities characteristic of FAS may reach as high as 5% in the USA and Western Europe (May et al., 2009). Some South African communities exhibit FAS/pFAS prevalences between 6.8 and 8.9% (May et al., 2007).

## Structural Brain Abnormalities in Fetal Alcohol Spectrum Disorders

### Brain Irregularities Associated with FASD

Autopsies of infants born with FASD paint a grim picture of the effects of alcohol on the brain. In these, the most severe cases of FASD, damage is ubiquitous throughout the brain (Riley and McGee, 2005). A general CNS disorganization is observed, with errors in neuronal migration, neuroglial heterotopias, microcephaly, and abnormalities of the brainstem, cerebellum, basal ganglia, hippocampus and corpus callosum, pituitary gland and optic nerve (Jones et al., 1973). The degree to which these characteristics are present in individuals that do not die during development or early childhood is unclear, but these findings are reflective of the widespread disruption of normal brain development that results from alcohol exposure.

Attempts to identify the degree of damage in living subjects with FASD have been carried out with magnetic resonance imaging (MRI). These reports indicate that individuals with FASD exhibit a reduction in the cranial vault as well as a corresponding decrease in the overall size of the brain (Mattson et al., 1992; Archibald et al., 2001). More recent reports indicate reduced gyrification of the cortex (Infante et al., 2015) and a reduction in the surface area of the anterior cingulate cortex (Migliorini et al., 2015) among adolescents with heavy prenatal alcohol exposure. Reduced activation of some cerebellar areas during rhythmic vs. non-rhythmic finger tapping (du Plessis et al., 2014) and global reductions in gray matter (Soh et al., 2015) are observed in subjects with FAS and FASD. Interestingly it appears that shrinkage is not uniform throughout the brain, but that certain areas such as the cerebellum and regions of the cortex are disproportionately affected (Archibald et al., 2001). The volume of the parietal lobe in the cerebral cortex is consistently reduced in FASD individuals (Archibald et al., 2001). Further, the composition of cortical brain regions is also impacted, with FASD brains exhibiting increases in gray matter, but reductions in white matter in the perisylvian cortices of the parietal and temporal lobes (Sowell et al., 2001). Such disruptions of normal cortical development are thought to underlie deficits in executive functions, verbal learning and recall, visuospatial processing and language present in individuals with FASD (Riley and McGee, 2005; Norman et al., 2009; Lebel et al., 2011).

The cerebellum is a primary target of alcohol effects; cerebellar volumes are significantly reduced and graded as with other deficits, with more severe shrinkage (>15%) present in subjects with FAS, and less loss among subjects with FASD that lack facial dysmorphology (Mattson et al., 1992; Archibald et al., 2001; Riley and McGee, 2005). Such disrupted cerebellar development is associated with deficiencies in balance, coordination, learning (eye-blink), verbal learning and memory and attention (Riley and McGee, 2005; Norman et al., 2009; Lebel et al., 2011).

The corpus callosum is another structure severely impacted by prenatal alcohol exposure. Agenesis (lack of formation) of the corpus callosum or anterior commissure, hypoplasia, volume reduction, heightened variability and displacement has been reported (Riley and McGee, 2005; Norman et al., 2009; Lebel et al., 2011). Due to consistent effects of *in utero* alcohol exposure on the corpus callosum, some have suggested that impaired formation of the corpus callosum may be a sensitive diagnostic indicator of prenatal exposure (Bookstein et al., 2002). White matter in general appears to be targeted by prenatal alcohol exposure, as diffusion tensor imaging and fractional anisotropy studies revealed white matter microstructural abnormalities and differences in white matter morphology in children and adolescents with FASD (Sowell et al., 2008).

Deficiencies in the basal ganglia have also been noted in subjects with FASD (Riley and McGee, 2005). Significant volume reductions are observed in the caudate of subjects with FASD (Archibald et al., 2001). Recent studies also suggest asymmetric formation of the caudate among subjects with either one trimester or all three trimesters of alcohol exposure *in utero* (Willford et al., 2010).

Unilateral decreases in hippocampal volume have been observed (Riley and McGee, 2005; Norman et al., 2009; Lebel et al., 2011). Scattered reports also suggest reduced hippocampal volume, though sometimes only when values are uncorrected for the reduced overall brain volume, or irregular brain shape associated with FASD (Joseph et al., 2014).

### Brain Irregularities in FASD Animal Models

To gain a greater understanding of alcohol effects on brain development and behavioral effects of prenatal alcohol exposure, several animal models have been developed. Models are present for nonhuman primates and sheep, which are advantageous due to gestation periods comparable to humans; however this is offset by substantially higher costs. Paralleling the human gestation period, the brain growth spurt in nonhuman primates and sheep occurs prenatally (Cudd, 2005). Other animal models have also been developed in *Drosphila*, zebrafish, guinea pigs and avian embryos (Fabregues et al., 1985; Cudd, 2005; Smith, 2008; McClure et al., 2011; Cole et al., 2012). The majority of FASD studies however, make use of mice and rats. Animal models of FASD target and expose animals to blood alcohol concentrations observed in humans during specific developmental hallmarks and then examine subsequent behavioral and structural changes induced by alcohol exposure (Table 1). These studies recapitulate several of the behavioral deficits observed in subjects with FASD in these animal models, with impairments observed in attention, inhibition, motor tasks, learning and social interactions. A full review of the behavioral consequences of alcohol in animal models of FASD was recently published (Patten et al., 2014).

**Table 1:** **Effects of alcohol exposure during critical neurodevelopmental periods**.

CNS developmental events	Consequences of alcohol exposure in animal models
Neural tube and crest formation	Craniofacial abnormalities Hypoplasia or aplasia of the corpus callosum Decrease in somatosensory neurons Malformation of the anterior cingulate cortex and hippocampus Anterior commissure, corpus callosum, and hippocampal commissure alterations and reduced myelination Sulik (2005), Miller (2007), and Cao et al. (2014)
CNS differentiation Neuronal migration Neurogenesis surge	Decreased neurogenesis Radial glia alterations Reduced migration and survival of neocortical, hippocampal and primary sensory neurons Miller (1986), Miller and Potempa (1990), Miller and Robertson (1993), and Sulik (2005)
Significant brain growth Myelination Dendritic arborization Synaptogenesis Gliogenesis (astrocytes and oligodendrocytes)	Neuronal apoptotic cell death Decreased complexity of dendritic arborization Behavioral impairment Microencephaly Apoptotic cell death of differentiating oligodendrocytes Delayed expression of myelin proteins Davies and Smith (1981), West et al. (1986), Bonthius and West (1990), Smith and Davies (1990), Ikonomidou et al. (2000), Granato et al. (2003), Cui et al. (2010), Hamilton et al. (2010), and Creeley et al. (2013)

The effects of alcohol are detrimental throughout the developing nervous system and therefore heavy alcohol exposure can be harmful to the embryo and fetus at any stage of gestation. At the structural level, rodent models of FASD exhibit similar brain alterations to those of human FASD subjects as with craniofacial abnormalities similar to FAS (Sulik, 2005), hypoplasia or aplasia of the corpus callosum (Qiang et al., 2002; Deng and Elberger, 2003; Sulik, 2005; Livy and Elberger, 2008), microencephaly (Bonthius and West, 1990), alterations of the neocortex (Miller, 1986, 2007; Miller and Potempa, 1990; Miller and Robertson, 1993; Mooney and Miller, 2007), hippocampus (Berman and Hannigan, 2000), cerebellum (Guerri, 1998; Luo, 2012) and basal ganglia (Creeley and Olney, 2013). Imaging studies of mouse brains also revealed reduced myelin in major midline white matter tracts after prenatal alcohol exposure (Cao et al., 2014).

Significant differentiation of the CNS occurs during GD 11–21 in rats and is highlighted by a burst of neurogenesis and population of the cerebral cortex and hippocampus by migrating neurons (Guerri, 1998). Neurogenesis during this time is wide-spread in the developing rat brain with the exception of the hippocampal dentate gyrus and granule cells of the cerebellum. These newborn cells then differentiate into neurons and glia and begin to mature, forming axonal and dendritic processes. With the direction of radial glia fibers, neurons of the cerebral cortex migrate from the germinal zone (Nadarajah and Parnavelas, 2002). Alcohol exposure during this period results in decreased neurogenesis and disrupted radial glia, as well as reduced migration and survival of neurons of the neocortex, hippocampus and principal sensory nucleus of the trigeminal nerve (Miller, 1986; Miller and Potempa, 1990; Miller and Robertson, 1993; Sulik, 2005).

Key maturation that occurs during the third trimester of gestation in humans instead takes place postnatally in rats. Exposure to alcohol during key developmental periods results in characteristic abnormalities consistent with the ongoing ontogenic events. A substantial increase in brain size, dendritic arborization and synaptogenesis, which corresponds to proliferation of astrocytes and oligodendrocyte precursors and initiation of myelination occurs from late gestation up to postnatal days (PNDs) 9 in rats (Bayer et al., 1993; Rice and Barone, 2000; Kelly et al., 2009). Alcohol exposure during the early postnatal period results in decreased neuronal number throughout the hippocampus (West et al., 1986), apoptotic cell death in several brain regions (Ikonomidou et al., 2000), microencephaly (Bonthius and West, 1990) and cerebellar anomalies (Bonthius and West, 1990). Prenatal and/or early postnatal alcohol exposure in rodents affects the structural plasticity of the brain as previously reviewed (Medina, 2011). Reduced dendritic arborization and dendritic spine density and complexity have been reported in hippocampal and neocortical pyramidal neurons after prenatal and/or postnatal alcohol exposure (Davies and Smith, 1981; Granato et al., 2003; Whitcher and Klintsova, 2008; Cui et al., 2010; Hamilton et al., 2010). This short overview does not cover the whole literature available on preclinical FASD studies but focuses on alcohol-induced anomalies that can be attributed to alterations in glial cells.

## Glial Contributions to Brain Development

### Radial Glia and Other Transient Glial Cells in the Developing Brain

Radial glia cells are widespread in the developing CNS where they differentiate directly from the neuroepithelial cells lining the ventricles. Radial glia exhibit processes that “radiate” in a directed, polarized fashion, thereby giving the cells their name. Radial glia are progenitors of neurons and several glial cell types, including astrocytes, oligodendrocytes and ependymal cells (Rowitch and Kriegstein, 2010). Radial glia are also essential for neuronal migration and the formation of the cerebral cortex. Indeed, radial glia have their cell body near the ventricular zone and extend their long process to the pial surface; newly differentiated post-mitotic neurons in the ventricular zone wrap around and migrate along the radial glia process to reach their location in the cortex (Rakic, 1972; Norris and Kalil, 1991). Radial glia persist in few specific locations in the adult brain (adult stem cells of the subventricular zone, Müller cells of the retina, and Bergman glia of the cerebellum; Sild and Ruthazer, 2011).

Midline glial cell populations that guide callosal axons are essential to the successful formation of the corpus callosum. Callosal axons extend toward and cross the midline in response to various molecular cues produced by midline glia cells. The glial wedge, one of the glial midline structures encountered by callosal axons, repels ipsilateral callosal axons towards the midline and guide them towards the contralateral cortex (Suárez et al., 2014). The glial wedge is composed of specialized astrocytes that develop from cortical radial glia between E13 and E17 (Shu et al., 2003). Defects in the glial wedge lead to the agenesis of corpus callosum (Chinn et al., 2015).

A transient midline raphe glial structure has been described (Van Hartesveldt et al., 1986), which is hypothesized to contribute to the development of the serotoninergic neurons of the raphe nuclei by providing trophic support for developing serotoninergic neurons through the release of glial-specific S100β, a growth factor for serotoninergic neurons (Azmitia et al., 1990), and by guiding neuronal migration (Van Hartesveldt et al., 1986).

### Astrocytes in Brain Development

The ratio of macroglia (astrocytes and oligodendrocytes) to neurons increases as the evolutionary complexity of the species increases (Sherwood et al., 2006), suggesting that astrocytes play an important role in higher order cognition and brain development. Indeed, recent studies indicate that astrocytes play a critical role in coordinating neuronal growth and migration with regulated secretion of extracellular matrix (ECM) proteins and trophic factors (Higgins et al., 1997; Pfrieger and Barres, 1997; Booth et al., 2000; Ullian et al., 2001; Martinez and Gomes, 2002; Yang et al., 2003; Christopherson et al., 2005; Pascual et al., 2005; Stellwagen and Malenka, 2006; Guizzetti et al., 2008). In addition, astrocytes are tuned to sense neuronal cues as evidenced by the diverse array of neurotransmitter receptors they express (Steinhauser and Kettenmann, 2009). As such, astrocytes are critical to proper neuronal circuit development.

The manner by which astrocytes and neurons communicate is still under heavy investigation. We recently identified a novel astrocyte-neuron interaction by which cholinergic activation leads to induction of multiple signaling cascades that enhance neurite outgrowth of hippocampal neurons (Guizzetti et al., 2008; Giordano et al., 2011). The majority of the factors secreted by astrocytes are proteases, protease inhibitors and ECM components (Moore et al., 2009). Following cholinergic stimulation of cultured astrocytes, we observed increases in ECM protein expression (fibronectin and laminin) both intracellularly and in the media and upregulation of factors that prevent degradation of the ECM (Guizzetti et al., 2008). Astrocyte-released proteins also play a major role in the regulation of synaptogenesis (reviewed in Guizzetti et al., 2014), the maintenance of brain homeostasis, neuroimmune function and blood-brain barrier development (Obermeier et al., 2013; Engelhardt and Liebner, 2014).

The brain is very rich in cholesterol (the brain contains 15–20% of the total body cholesterol, but represents only about 5% of the total body weight); cholesterol is necessary for proper brain health and development (Martín et al., 2014). Cholesterol circulates through the brain in association with astrocyte-produced lipoproteins (Martín et al., 2014). Both microglia and astrocytes express apolipoprotein E (apoE), which is a major component of brain lipoproteins (Diedrich et al., 1991; Nakai et al., 1996). The process by which cholesterol is removed from the brain is important for overall brain health and homeostasis (Dietschy, 2009).

Brain lipoproteins generally exert positive effects on CNS functions and are protective against neurodegenerative diseases. Astrocyte-produced lipoproteins, however, also induce a net efflux of cholesterol from astrocytes and neurons (Guizzetti et al., 2007; Kim et al., 2007; Chen et al., 2013). During development, cholesterol is essential for several brain functions including cell proliferation, neuronal survival, and activation of the sonic hedgehog pathway. Genetic defects in cholesterol synthesizing enzymes, such as the one causing Smith-Lemli-Opitz-Syndrome (SLOS), result in low levels of cholesterol in all the tissues, and are associated with altered brain development, intellectual disability and behavioral disorders (Nowaczyk et al., 1999). Therefore, increased brain lipoproteins may be deleterious to the developing brain because it may reduce brain cholesterol levels (Guizzetti and Costa, 2007).

### Oligodendrocytes and Myelination in Brain Development

Myelin is formed by the plasma membrane of oligodendrocytes (in the CNS) or Schwann cells (in the peripheral nervous system) wrapping around axon segments many times (Szuchet et al., 2015) and forming a compacted insulating sheath, that serves to improve the speed and efficiency of electrical signal conduction along the myelinated axon. Myelination is a dynamic process that can be modulated by the axonal release of neurotransmitter and by environmental factors. Oligodendrocytes and their myelin sheaths also provide supportive factors to maintain axonal health (Rosenbluth, 1999; Simons and Trotter, 2007; Fields, 2010; Nave, 2010; Mitew et al., 2013; Nualart-Marti et al., 2013).

Developmentally, myelination occurs as one of the final stages of brain development. In humans, most myelination takes place during the first 20 years of postnatal life (Lebel et al., 2008). While the bulk of myelination occurs in relatively early postnatal life, it is now clear that myelination continues throughout life (Bartzokis et al., 2012; Young et al., 2013). Coinciding with their later development, oligodendrocytes are the final cells of the CNS to mature. Nevertheless, oligodendrocyte precursor cells (OPCs) are present at embryonic day 12.5 in mice. Production of OPCs is localized to distinct areas, thus, migration of this cell type throughout the brain is an important phase of development. OPCs are generated throughout life and may contribute to myelination in adulthood (El Waly et al., 2014). Genetic influences appear to strongly regulate myelination, however, mounting evidence suggests that experiential factors also influence myelination. Control of oligodendrocyte differentiation and myelination occurs through a diverse array of transcription factors, extracellular signals and intracellular pathways (Mitew et al., 2013).

### Microglia in Normal Brain Development

Microglia are of hematopoietic origin and are the resident “professional” immune cell, composing roughly 10% of all cells in the brain (Benarroch, 2013). Under normal conditions, microglia exhibit a ramified morphology, allowing them to sample and survey from the environment for signs of infection or insult. Upon activation, microglia become rod-like or amoeboid, with a multinucleated or epithelioid appearance (Benarroch, 2013). Activated microglia also exhibit changes in gene expression, function and produce an array of pro-inflammatory cytokines, chemokines and reactive oxygen species (ROS). Microglia polarize, much like T-cells, becoming relatively pro- or anti-inflammatory depending on the surrounding environmental cues present (Nakagawa and Chiba, 2015). Microglia are also capable of clearing pathogens or cellular debris via phagocytosis. Under normal conditions, microglia are important contributors to maintaining brain homeostasis, but under pathological conditions, microglia can become chronically activated, inducing inflammation and neurodegeneration when functionally impaired (Saijo and Glass, 2011).

During embryonic development in rodents, microglia appear in the activated state with an amoeboid shape and transition to a resting ramified morphology shortly after birth. Microglia also play a major role during brain development. Indeed, microglia phagocytize neural precursor cells, particularly during late stages of cortical neurogenesis therefore regulating the size of the neural precursor cell pool in the developing cortex (Cunningham et al., 2013). Microglia also instruct neuronal apoptosis, engulf and phagocytize apoptotic neurons (Bessis et al., 2007; Marín-Teva et al., 2011) and induce synaptic pruning (Paolicelli et al., 2011).

## Glial Dysfunction in Fasd

### Effects of Alcohol on Radial Glia and Other Glial Structures in the Developing Brain

Heavy prenatal alcohol exposure alters neuronal migration and induces glial heterotopia (Jones et al., 1973). Radial glia are targets of alcohol damage and are critical to proper neuronal migration. Radial glia cells derived from 13-day rat fetuses exhibited reductions in GFAP, but not vimentin expression both *in vivo* and in culture (Valles et al., 1996). Prenatal alcohol exposure *in vivo* also causes a decrease in GFAP fibers in cerebellar Bergman glia on PND 15, reflecting a delayed maturation of these cells that may contribute to the delayed migration of granule cells (Shetty and Phillips, 1992). Developmental exposure to alcohol lead to decreased density and fasciculation of radial glial processes that run from the ventricular surface to the pial surface (Miller and Robertson, 1993) during the first week following birth. This was accompanied by a decrease in vimentin staining on PND 5 compared to non-exposed control animals. Chronic exposure to alcohol via liquid diet led to decreased cell division in radial glia cultured from E12 telencephalon, and also reduced the number of multipotent progenitor cells derived from neurospheres, with concurrent decreases in the progenitor-maintaining proteins Notch1 and fibroblast growth factor 2 (Rubert et al., 2006). This study also observed a greater percentage of cells derived from alcohol-exposed embryos remaining as radial glia in culture as opposed to differentiating into neurons or astrocytes following 2 days of culture compared to control cultures (Rubert et al., 2006). Alcohol exposure damages neural progenitor cells, limiting their survival and impeding their differentiation into astrocytes (Taléns-Visconti et al., 2011; Nash et al., 2012). Culture models using neurospheres also demonstrate that selective exposure of neural precursor cells to alcohol impairs cell division and astrocyte formation (Vemuri and Chetty, 2005). Thus, alcohol exposure disrupts the development and maturation of radial glia cells that act both as precursor cells for neurons, astrocytes and oligodendrocytes, as well as aid in the migration of other progenitor cells throughout the developing CNS (Table 2).

**Table 2:** **Cell specific consequences of alcohol exposure**.

Cell type	Consequences of developmental alcohol exposure
Radial Glia	Reduced GFAP expression Decreased density and fasciculation of processes Reduced cell division Decreased density of multipotent progenitor cells and associated factors Impaired differentiation into astrocytes Miller and Robertson (1993), Valles et al. (1996), Vemuri and Chetty (2005), Rubert et al. (2006), Taléns-Visconti et al. (2011), and Nash et al. (2012)
Oligodendrocytes	Reduced and disorganized white matter Delayed myelination Decreased myelin thickness Ultrastructural anomalies in myelin Altered myelin biochemical profile Decreased oligodendrocyte differentiation Delayed and reduced expression of key myelin proteins Cell death Druse and Hofteig (1977), Hofteig and Druse (1978), Gnaedinger et al. (1984), Samorajski et al. (1986), Phillips and Krueger (1992), Lancaster (1994), Phillips (1994), Zoeller et al. (1994), Parson et al. (1995), Riley et al. (1995), Pinazo-Duran et al. (1997), Archibald et al. (2001), Guerri et al. (2001), Dalitz et al. (2008), Sowell et al. (2008), Bichenkov and Ellingson (2009), and Creeley et al. (2013)
Astrocytes	Reduced proliferation and survival of progenitor cells Decreased production and release of neurotrophic factors Increased production and release of inhibitors of neurite outgrowth Activation of inflammatory signaling pathways (TLR-4, nitric oxide (NOS), cyclooxygenase 2) Decreased production of the primary brain antioxidant glutathione Disruption of cholesterol homeostasis Reduced astrocyte differentiation Altered expression of GFAP Altered production of extracellular matrix proteins Davies and Smith (1981), Renau-Piqueras et al. (1989), Miller and Potempa (1990), Saez et al. (1991), Fletcher and Shain (1993), Goodlett et al. (1993), Goodlett et al. (1997), Lokhorst and Druse (1993), Resnicoff et al. (1994), Montoliu et al. (1995), Guizzetti and Costa (1996), Vallés et al. (1997), Kötter and Klein (1999), Granato et al. (2003), Blanco et al. (2004, 2005), Tomás et al. (2005), Watts et al. (2005), Rathinam et al. (2006), Guizzetti et al. (2007, 2011), Pascual and Guerri (2007), Whitcher and Klintsova (2008), Cui et al. (2010), Hamilton et al. (2010), Zhou et al. (2014), and Topper et al. (2015)
Microglia	Increased phagocytosis Reduced survival Increased production of inflammatory mediators and oxidative stress Increase in microglia-mediated hypothalamic neuron death Decreased production of neurotrophic factors Boyadjieva and Sarkar (2010), Boyadjieva and Sarkar (2013a,b), and Kane et al. (2011)

The formation of the corpus callosum is strongly affected by prenatal alcohol exposure (Riley and McGee, 2005; Norman et al., 2009; Lebel et al., 2011). While no studies have investigated the effect of alcohol on midline glial cell populations directing the formation of the corpus callosum, such as the glial wedge, it is possible to speculate that alcohol may affect the release or expression of guiding molecules by these cells.

Interestingly, the number of glial cells in transient midline raphe glial structures expressing S100β, a trophic factor for serotoninergic neurons (Azmitia et al., 1990), is decreased. Expression of S100β is reduced in the midline raphe of prenatal alcohol exposed rats and mice (Eriksen et al., 2000; Zhou et al., 2001; Tajuddin et al., 2003), which may underlie the reduced number of serotoniergic neurons as well their migration and development observed in FASD animal models (Tajuddin and Druse, 1999, 2001; Zhou et al., 2001).

### Role of Astrocytes in FASD

#### Glial Fibrillary Acidic Protein (GFAP) Expression

Glial fibrillary acidic protein (GFAP), an intermediate filament often used as a marker for astrocytes whose upregulation is considered a marker for reactive astrogliosis, and has been investigated in several studies in relation to FASD. Astrocytes prepared from fetuses exposed *in vivo* to alcohol and maintained in culture in the presence of alcohol present decreased GFAP expression and failed to develop processes (Renau-Piqueras et al., 1989). When astrocyte cultures are exposed to alcohol, GFAP levels increase initially after which GFAP expression decreases after 21 days in culture (Saez et al., 1991). A decrease in GFAP expression was confirmed *in vivo* after prenatal alcohol exposure in the brain of postnatal animals and was attributed to increased DNA methylation in the GFAP promoter region (Vallés et al., 1997).

Neonatal alcohol exposure lead to an increase in GFAP expression in the cortex and hippocampus when exposure was carried out via artificial rearing (Fletcher and Shain, 1993; Goodlett et al., 1993) and an increase in GFAP in the parietal cortex after alcohol exposure via intragastric intubation (Goodlett et al., 1997), but no GFAP upregulation was observed when alcohol was administered via vapor inhalation (Ryabinin et al., 1995). More recently, neonatal alcohol exposure via vapor inhalation has been shown to increase GFAP expression in the cerebellum and hippocampus during the alcohol-withdrawal period (Topper et al., 2015). From these reports it can be concluded that the reduction of GFAP levels observed *in vitro* is recapitulated by *in vivo* experiments when alcohol is administered prenatally. However, the effect of neonatal exposure to alcohol on GFAP levels is not conclusive. Studies suggest that the effects of alcohol on astrocyte GFAP levels vary depending on the developmental stage of astrocytes at the time of exposure, on alcohol levels, route of administration, length of the treatment, and alcohol-withdrawl status.

The fact that, when observed, the increase in GFAP expression is not generalized to the whole brain, but rather confined to specific areas, suggests that this may not be a direct effect of alcohol on astrocytes, but rather a secondary effect caused, for instance, by increased neuronal damage, release of proinflammatory cytokines by microglia, or damage to blood brain vessels. However, the question remains regarding the significance of changes in alcohol-induced GFAP expression in astrocytes and whether these changes play a positive (protective) or negative role in alcohol-induced developmental brain injury. Several studies during the last 20 years have shown that astrogliosis is not a simple “all or nothing” response; astrocytes are capable of a spectrum of changes (ranging from reversible alterations in gene expression and cellular hypertrophy to cell proliferation, scar formation and permanent tissue rearrangement) tailored to the type of insult they are responding to Sofroniew (2014).

#### Astrocyte Proliferation

Exposure of primary astrocyte cultures to alcohol also impairs astrocyte proliferation induced by serum, IGF-1, the cholinergic agonist carbachol, and PDGF (Resnicoff et al., 1994; Guizzetti and Costa, 1996; Kötter and Klein, 1999). Alcohol appears to affect astrocyte proliferation by inhibiting specific signal transduction pathways. For instance, we have reported that alcohol selectively inhibits carbachol-stimulated phospholipase D signaling (inducing the formation of the second messenger phosphatidic acid, which activates Akt, p70S6K and PKCζ) in astrocytes, leaving unaffected other pathways activated by carbachol (Guizzetti and Costa, 2000, 2002; Guizzetti et al., 2003, 2004, 2014; Tsuji et al., 2003). Prenatal alcohol exposure has been reported to cause a decrease in astrocyte number (Miller and Potempa, 1990), an observation that supports the *in vitro* studies.

#### Astrocyte-Induced Neuronal Plasticity

*In vivo* pre- and post-natal alcohol exposure affects neuronal structural plasticity (Medina, 2011). Alcohol strongly affects the release of neuritogenic factors by astrocytes (Tomás et al., 2005). Reduced dendritic arborization and dendritic spine density and complexity is reported in hippocampal and neocortical pyramidal neurons (Davies and Smith, 1981; Granato et al., 2003; Whitcher and Klintsova, 2008; Cui et al., 2010; Hamilton et al., 2010) and may be due to alcohol-induced alterations in the secretion of factors modulating neuronal development and plasticity by astrocytes. A reduction in neuritogenesis and neuronal survival was reported in naïve neurons co-cultured with astrocytes prepared from alcohol-exposed rats (Pascual and Guerri, 2007). In an earlier study, neurons cultured in conditioned media from astrocytes exposed in culture to 100 mM alcohol for 4 days displayed reduced DNA content, neurite length, number of serotonin neurons and serotonin uptake (Lokhorst and Druse, 1993). Astrocytes also modulate the effect of alcohol on dendrite development in culture (Yanni et al., 2002). Astrocyte-specific expression of serum response factor in a ferret model of early alcohol exposure reverses alcohol-induced reductions in ocular dominance plasticity (Paul and Medina, 2012).

We have reported that alcohol exposure inhibits the ability of carbachol-treated astrocytes to foster neurite outgrowth in neurons co-cultured with astrocytes after treatment by decreasing the release of neuritogenic proteins laminin and fibronectin (Guizzetti et al., 2010), an effect reproduced in hippocampal slices (Giordano et al., 2011). This effect is in part mediated by the upregulation of the tissue-type plasminogen activator (tPA) which converts plasminogen to plasmin, an extracellular proteolytic enzyme that degrades the ECM (Zhang et al., 2014). Chondroitin sulfate proteoglycans (CSPGs) are inhibitors of neurite outgrowth. We found that alcohol upregulates the levels of CSPGs through the inhibition of the enzyme arulsulfatase B (ARSB). ARSB degrades the chondroitin sulfate moiety of CSPGs and leads to the extracellular proteolysis of the core-protein in astrocyte cultures *in vitro* and after neonatal alcohol exposure *in vivo* (Zhang et al., 2014).

#### Oxidative Stress

Exposure of astrocyte cultures to alcohol leads to reductions in glutathione (GSH), and induction of cyclooxygenase 2 (Cox-2) as a result of NF-κB signaling, and the formation of ROS in astrocytes (Montoliu et al., 1995; Blanco et al., 2004). The induction of reactive species and inflammatory signaling appears to be mediated via toll-like receptor 4 (TLR4) and interleukin one receptor (IL-1R1) signaling in astrocytes, as inhibition of these receptors prevents alcohol-induced AP-1 and NF-κB activation as well as induction of nitric oxide (NOS) and Cox-2 (Blanco et al., 2005). Cortical neurons undergo apoptotic cell death in response to depletion of GSH and ROS production following alcohol exposure. Co-culturing neurons with astrocytes decreased GSH depletion and attenuated neuronal death following alcohol treatment (Watts et al., 2005; Rathinam et al., 2006).

#### Brain Lipid Homeostasis

Cholesterol homeostasis is maintained by astrocyte-released lipoproteins that can extract cholesterol from astrocytes and neurons. Our studies indicate that alcohol disrupts this process by increasing release of lipoprotein from astrocytes which leads to increased efflux of cholesterol from astrocytes and neurons (Guizzetti et al., 2011) and may lead to cholesterol-loaded lipoproteins exiting the brain and subsequent reductions in cholesterol (Zhou et al., 2014). The transporter ABCA1 (ATP binding cassette-A1) is essential for the generation of nascent lipoproteins in astrocytes. Cholesterol efflux is mediated by ABCG1 and ABCG4 transporters and leads to the lipidation and remodeling of nascent, lipid poor lipoproteins (Koldamova et al., 2003; Hirsch-Reinshagen et al., 2004; Wahrle et al., 2004). Our studies indicate that alcohol upregulates expression of ABCA1 and ABCG1 in astrocyte cultures, thereby increasing cholesterol efflux and reducing brain cholesterol levels (Guizzetti et al., 2007). Astrocyte-released lipoproteins also increase cholesterol efflux from neurons through a mechanism likely mediated by ABCG4 (Zhou et al., 2014). *In vivo*, neonatal alcohol exposure increases cortical levels of ABCA1 (Guizzetti et al., 2007); while, prenatal alcohol exposure up-regulates ABCA1 and ABCG1 and reduces the levels of cholesterol in the neocortex of GD 21 females (Zhou et al., 2014).

### Role of Oligodendrocytes in FASD

Examination of the impact of alcohol on oligodendrocytes and by extension, myelination has been intermittent at best, with the bulk of studies occurring in the late 1970s or early 1990s. White matter (myelin/oligodendrocytes) is another target of the developmental effects of alcohol as evidenced by lower volumes and sometimes complete lack of formation of major white matter tracts in the brain of individuals with FASD (Table 2; Riley et al., 1995; Sowell et al., 2008). With the availability and affordability of advanced imaging techniques, interest is once again growing and high resolution examination of myelin in FASD subjects is now possible.

As previously described, at first glance, prenatal exposure to alcohol may not be expected to exert a substantial effect on myelination in adults, given that much of the myelination that occurs in humans happens long after birth. Nevertheless, developmental exposure to alcohol permanently impacts the programming of OPCs, as imaging studies indicate widespread anomalies in children and adults with FASD (Archibald et al., 2001; Sowell et al., 2008). These findings are recapitulated in rat models of FASD, where alcohol slows myelination and disrupts the myelin ultrastructure (Lancaster, 1994; Phillips, 1994; Pinazo-Duran et al., 1997).

Developmental alcohol exposure in rats causes clear changes in myelin, with a distinct shift in the expression patterns of oligodendrocytes (Druse and Hofteig, 1977; Hofteig and Druse, 1978; Gnaedinger et al., 1984). The greatest harm to myelin and oligodendrocytes is associated with exposure during the first 10 PNDs in animal models, and is characterized by myelin malformation and morphological deficits in oligodendrocytes (reviewed in Guizzetti et al., 2014). Studies in sheep suggest that third-trimester alcohol exposure leads to myelin malformation and morphological deficits in oligodendrocytes (Dalitz et al., 2008). Myelin basic protein (MBP), which as its name implies is a critical element of the myelin sheath, is delayed and reduced in its expression following postnatal alcohol exposure in the cerebellum of PND 15 rats (Zoeller et al., 1994). Exposure of cultured oligodendrocytes to alcohol also leads to a decrease in MBP levels (Bichenkov and Ellingson, 2009). Monkeys exposed to alcohol during late stage development exhibit extensive apoptosis of oligodendrocytes in white matter regions (Creeley et al., 2013), further highlighting oligodendrocytes as targets of alcohol in the developing brain. Studies in primary mouse oligodendrocyte cultures suggest that acetaldehyde, the toxic, metabolic byproduct of alcohol is highly lethal to oligodendrocytes, while very high concentrations of alcohol are necessary to cause damage (Coutts and Harrison, 2015).

A common characteristic of individuals with FASD is impaired visual function (Strömland, 2004). Myelination of the optic nerve is disrupted in animal models of FASD, with reports of decreased thickness, as well as aberrant and fewer myelin sheaths (Samorajski et al., 1986; Phillips and Krueger, 1992; Parson et al., 1995; Pinazo-Duran et al., 1997). Developmental alcohol exposure interferes with oligodendrocyte maturation and delays expression of MBP resulting in ultrastructural harm to the myelin sheaths and a decreased number of myelinated optic nerve axons (Guerri et al., 2001). Such alterations in myelin may be responsible for the hypoplasia of the optic nerve present in FAS.

This data suggests that myelin and more specifically, oligodendrocyte development is a target of alcohol effects in the developing brain and that early exposure can lead to persistent, life-long deficiencies. Disruptions in survival and maturation of oligodendrocytes are likely to impair the formation of neurocircuitry and the efficient conduction of neuronal signals. Future studies are needed, however, to elucidate the effects of alcohol on oligodendrocytes and OPCs to understand the pathways that lead to alcohol-induced dysfunction.

### Role of Microglia in FASD

Microglia possess an assortment of receptors designed to detect aberrant “danger” signals so that an appropriate response can be mounted. Recent work indicates that alcohol can induce microglial activation (Fernandez-Lizarbe et al., 2009) and even death (Table 2; Kane et al., 2011). Initial studies suggest this activation may occur via the Bcl-2 associated X Protein (BAX), as mice lacking BAX exhibit blunted microglial activation and pro-inflammatory signaling following alcohol exposure on PND 7 or PND 8 (Ahlers et al., 2015). In cultures, alcohol increases the release of inflammatory cytokines from microglia and reduces intracellular cAMP and brain-derived neurotrophic factor in co-cultures of hypothalamic neurons and microglia (Boyadjieva and Sarkar, 2010, 2013a). Oxidative stress is also increased in alcohol exposed microglia (Boyadjieva and Sarkar, 2013b). Alcohol exposure also affects the viability of microglia both *in vitro* and *in vivo* following neonatal alcohol exposure (Kane et al., 2011). The response of microglia to inflammatory factors is variable, with repeated exposures leading to a sensitized response (Perry and Teeling, 2013). Some have hypothesized that prenatal alcohol exposure may lead to long-term sensitization of microglia that results in persistent inflammatory signaling in the brain following insult (Chastain and Sarkar, 2014). This priming hypothesis is consistent with a study observing increased inflammatory signaling in prenatally-exposed rats in a model of rheumatoid arthritis in adult animals (Zhang et al., 2012). Recent studies suggest that alcohol-induced microglial activation can be reduced by treatment with peroxisome proliferator-activated receptor γ agonists such as pioglitazone (Drew et al., 2015), thus identifying a potential treatment.

Alterations in microglial function also impact neuronal health. Microglia-conditioned media contributes to alcohol-induced apoptosis in immature hypothalamic neurons (Boyadjieva and Sarkar, 2010, 2013a,b). Neonatal alcohol exposure in rodents induces neurotoxicity in hypothalamic neurons *in vivo*, which appears to be mediated, at least in part, by microglia (Sarkar et al., 2007). Several excellent reviews offer greater depth and discussion of alcohol effects on microglia and the subsequent developmental impacts (Drew and Kane, 2013, 2014; Chastain and Sarkar, 2014; Crews and Vetreno, 2014).

### Glial Cells in Abnormal Brain Development

FASD is not the only neurodevelopmental disorder for which glial dysfunction is a major contributing factor. Rett syndrome is a neurodevelopmental disorder characterized by seizures, motor impairment, neurogenic apneas and delayed or absent speech (Chahrour and Zoghbi, 2007). Mutations in the methyl-CpG-binding protein 2 (*MeCP2*) gene appear to be a critical factor in the disease. Interestingly, *MeCP2* is abundantly expressed in neurons, but also present in astrocytes, microglia and oligodendrocytes. Original theories regarding the disease focused almost exclusively on neurons, but recent evidence now indicates that glial cells (astrocytes, microglia and oligodendrocytes) are important contributors to disease pathology (reviewed in Guizzetti et al., 2014). Disruptions in astrocyte development and function may contribute to autism, fragile X, Down syndrome, Costello syndrome, neurofibromatosis-1, Noonan syndrome and Cardiofaciocutaneous syndrome (Garcia et al., 2010; Jacobs and Doering, 2010; Sloan and Barres, 2014). Astrocyte dysfunction is also hypothesized to partially underlie the neurodevelopmental origins of major depressive disorder (MDD) and schizophrenia as evidenced by glial anomalies in patients with these disorders (Sloan and Barres, 2014). Neurotoxicants such as diazinon, an organophosphate insecticide, and its active metabolite diazoxon, as well as manganese impair the development of hippocampal pyramidal neurons when they are co-cultured with astrocytes previously treated with these compounds (Giordano et al., 2009; Pizzurro et al., 2014). There are also genetic mutations that interfere with normal astrocyte function, such as the gain of function mutation in the GFAP gene thought to be responsible for Alexander’s disease. Alexander’s disease is characterized by atypical myelination, developmental delay, and macroencephaly, with astrocytes exhibiting Rosenthal fiber accumulation, and abnormally high expression of GFAP (Quinlan et al., 2007; Messing et al., 2012). The disruption of myelination highlights cross-talk that occurs between astrocytes and oligodendrocytes during neurodevelopment. Prenatal infections lead to activation of microglia and astrocytes and are associated with decreased oligodendrocyte density, abnormal myelination, and schizophrenia (Anderson and Maes, 2013; Meyer, 2013). Microglial activation has been observed in multiple brain regions of autism spectrum disorder patients (Morgan et al., 2010; Rodriguez and Kern, 2011; Suzuki et al., 2013). Prenatal stressors such as vitamin B12 deficiency, restraint stress, hypoxia, opioids and methamphetamine result in compromised myelination in the postnatal brains of exposed mothers (Lövblad et al., 1997; Baud et al., 2004; Melo et al., 2008; Sanchez et al., 2008). In conclusion, glial cells are important contributors to pathological events that may occur in the brain during development and therefore have great potential as therapeutic targets for the treatment of neurodevelopmental diseases.

## Conclusions and Future Research Directions

Several of the structural abnormalities observed in FASD and in FASD animal models are consistent with altered glial cell function. Defects in neuronal migration observed in FASD are likely due to reported effects of alcohol on radial glia. Prenatal alcohol can also induce agenesis or hypoplasia of the corpus callosum, which may be due to an effect of alcohol on midline glial populations causing them to alter the signals these cells send to the axons that need to cross the midline into the opposite hemisphere. Studies on the effect of alcohol on these structures have not been carried out, but may represent a new, interesting avenue of investigation.

Recent imaging studies of FASD children show defects in white matter fiber tracts that may arise from the reported effects of alcohol on oligodendrocytes or their precursors. Similarly, alcohol exposure during the brain growth spurt and the resultant microencephaly that occurs also highlights the glial effects of alcohol. There is evidence that reduced dendritic arborization and structural plasticity observed in FASD models may be due, at least in part, to changes in factors released by astrocytes, which are known to have a major role in neuronal development and brain maturation. Finally, the neuronal loss reported in several FASD models may be caused by reduced release of trophic factors and antioxidants by astrocytes or increased release of neuroinflammatory molecules by microglia and astrocytes.

A major challenge of glia and FASD research is to causally link the effects observed in glial cells to neuronal abnormalities in the brain. *In vitro* studies have been instrumental in demonstrating the concept that, by affecting glial cell functions, alcohol alters neuronal survival and development. However, these studies need to be validated *in vivo* and to be linked to behavioral outcomes. Molecular approaches for manipulating glial cell signaling and functions *in vivo* (for instance, Xie et al., 2015) and methods for *ex-vivo* isolation of specific brain cell populations are becoming available and should be used in FASD research.

The role of sex in susceptibility of glial cells to the consequences of fetal alcohol exposure is almost entirely unexplored, although differences have been observed in the function of astrocytes derived from male and female animals in culture (Liu et al., 2008; Santos-Galindo et al., 2011; Wilhelm et al., 2015). Individuals with FASD exhibit different deficits in eye movement depending on whether they are male or female (Paolozza et al., 2015). Sex differences in FASD preclinical models have been reported in a number of instances; for instance, developmental alcohol exposure leads to social avoidance in females, but increased play fighting in males in a model of FASD (Varlinskaya and Mooney, 2014); females exposed to prenatal alcohol and chronic mild stress exhibit increases in learned helplessness, and disrupted social interactions (Hellemans et al., 2010), sexually dimorphic effects of prenatal alcohol on the development of the hypothalamic-pituitary-adrenal axis have been extensively documented (Weinberg et al., 2008); sex differences in spatial learning deficits after neonatal alcohol exposure have also been reported (Kelly et al., 1988; Goodlett and Peterson, 1995). The hypothesis that glial cells in males vs. females are differentially affected by alcohol during brain development is worth further examination.

We have summarized published evidence that strongly support the notion that alcohol detrimentally affects the glial cells of the developing brain and that disruption of the function of these cells leads to neuronal deficits. It is through interactions between neurons and glial cells that the CNS is successfully developed. Thus, elucidating not just the cell-specific effects of alcohol, but also the cell-cell interactions that occur is necessary to fully understand and effectively treat abnormal brain development. Unraveling the complex biological disruptions induced by fetal alcohol exposure and identifying causal links between glial dysfunction and brain structural and functional abnormalities as well as behavioral impairments is an important next step to identifying optimal targets for therapeutic development.

## Conflict of Interest Statement

The authors declare that the research was conducted in the absence of any commercial or financial relationships that could be construed as a potential conflict of interest.

## References

[B1] Abel E. L. Sokol R. J. (1987). Incidence of fetal alcohol syndrome and economic impact of FAS-related anomalies. Drug Alcohol Depend. 19, 51–70. 10.1016/0376-8716(87)90087-1 3545731

[B2] Ahlers K. E. Karaçay B. Fuller L. Bonthius D. J. Dailey M. E. (2015). Transient activation of microglia following acute alcohol exposure in developing mouse neocortex is primarily driven by BAX-dependent neurodegeneration. Glia 63, 1694–1713. 10.1002/glia.22835 25856413 PMC4534325

[B3] Anderson G. Maes M. (2013). Schizophrenia: linking prenatal infection to cytokines, the tryptophan catabolite (TRYCAT) pathway, NMDA receptor hypofunction, neurodevelopment and neuroprogression. Prog. Neuropsychopharmacol. Biol. Psychiatry 42, 5–19. 10.1016/j.pnpbp.2012.06.014 22800757

[B4] Archibald S. L. Fennema-Notestine C. Gamst A. Riley E. P. Mattson S. N. Jernigan T. L. (2001). Brain dysmorphology in individuals with severe prenatal alcohol exposure. Dev. Med. Child Neurol. 43, 148–154. 10.1111/j.1469-8749.2001.tb00179.x 11263683

[B5] Azmitia E. C. Dolan K. Whitaker-Azmitia P. M. (1990). S-100B but not NGF, EGF, insulin or calmodulin is a CNS serotonergic growth factor. Brain Res. 516, 354–356. 10.1016/0006-8993(90)90942-5 2194631

[B6] Bartzokis G. Lu P. H. Heydari P. Couvrette A. Lee G. J. Kalashyan G.. (2012). Multimodal magnetic resonance imaging assessment of white matter aging trajectories over the lifespan of healthy individuals. Biol. Psychiatry 72, 1026–1034. 10.1016/j.biopsych.2012.07.010 23017471

[B7] Baud O. Daire J. L. Dalmaz Y. Fontaine R. H. Krueger R. C. Sebag G.. (2004). Gestational hypoxia induces white matter damage in neonatal rats: a new model of periventricular leukomalacia. Brain Pathol. 14, 1–10. 10.1111/j.1750-3639.2004.tb00492.x 14997932 PMC8095946

[B8] Bayer S. A. Altman J. Russo R. J. Zhang X. (1993). Timetables of neurogenesis in the human brain based on experimentally determined patterns in the rat. Neurotoxicology 14, 83–144. 8361683

[B9] Benarroch E. E. (2013). Microglia: multiple roles in surveillance, circuit shaping and response to injury. Neurology 81, 1079–1088. 10.1212/WNL.0b013e3182a4a577 23946308

[B10] Berman R. F. Hannigan J. H. (2000). Effects of prenatal alcohol exposure on the hippocampus: spatial behavior, electrophysiology and neuroanatomy. Hippocampus 10, 94–110. 10.1002/(SICI)1098-1063(2000)10:1<94::AID-HIPO11>3.0.CO;2-T 10706221

[B11] Bessis A. Béchade C. Bernard D. Roumier A. (2007). Microglial control of neuronal death and synaptic properties. Glia 55, 233–238. 10.1002/glia.20459 17106878

[B12] Bichenkov E. Ellingson J. S. (2009). Ethanol alters the expressions of c-Fos and myelin basic protein in differentiating oligodendrocytes. Alcohol 43, 627–634. 10.1016/j.alcohol.2009.09.026 20004340

[B13] Blanco A. M. Pascual M. Valles S. L. Guerri C. (2004). Ethanol-induced iNOS and COX-2 expression in cultured astrocytes via NF-κB. Neuroreport 15, 681–685. 10.1097/00001756-200403220-00021 15094475

[B14] Blanco A. M. Vallés S. L. Pascual M. Guerri C. (2005). Involvement of TLR4/type I IL-1 receptor signaling in the induction of inflammatory mediators and cell death induced by ethanol in cultured astrocytes. J. Immunol. 175, 6893–6899. 10.4049/jimmunol.175.10.6893 16272348

[B15] Bonthius D. J. West J. R. (1990). Alcohol-induced neuronal loss in developing rats: increased brain damage with binge exposure. Alcohol. Clin. Exp. Res. 14, 107–118. 10.1111/j.1530-0277.1990.tb00455.x 1689970

[B16] Bookstein F. L. Sampson P. D. Connor P. D. Streissguth A. P. (2002). Midline corpus callosum is a neuroanatomical focus of fetal alcohol damage. Anat. Rec. 269, 162–174. 10.1002/ar.10110 12124903

[B17] Booth G. E. Kinrade E. F. Hidalgo A. (2000). Glia maintain follower neuron survival during Drosophila CNS development. Development 127, 237–244. 10603342 10.1242/dev.127.2.237

[B18] Boyadjieva N. I. Sarkar D. K. (2010). Role of microglia in ethanol’s apoptotic action on hypothalamic neuronal cells in primary cultures. Alcohol. Clin. Exp. Res. 34, 1835–1842. 10.1111/j.1530-0277.2010.01271.x 20662807 PMC2965273

[B19] Boyadjieva N. I. Sarkar D. K. (2013a). Cyclic adenosine monophosphate and brain-derived neurotrophic factor decreased oxidative stress and apoptosis in developing hypothalamic neuronal cells: role of microglia. Alcohol. Clin. Exp. Res. 37, 1370–1379. 10.1111/acer.12104 23550806 PMC3706564

[B20] Boyadjieva N. I. Sarkar D. K. (2013b). Microglia play a role in ethanol-induced oxidative stress and apoptosis in developing hypothalamic neurons. Alcohol. Clin. Exp. Res. 37, 252–262. 10.1111/j.1530-0277.2012.01889.x 22823548 PMC3481017

[B21] Cao W. Li W. Han H. O’Leary-Moore S. K. Sulik K. K. Allan Johnson G.. (2014). Prenatal alcohol exposure reduces magnetic susceptibility contrast and anisotropy in the white matter of mouse brains. Neuroimage 102(Pt. 2), 748–755. 10.1016/j.neuroimage.2014.08.035 25175539 PMC4252734

[B22] Chahrour M. Zoghbi H. Y. (2007). The story of Rett syndrome: from clinic to neurobiology. Neuron 56, 422–437. 10.1016/j.neuron.2007.10.001 17988628

[B23] Chastain L. G. Sarkar D. K. (2014). Role of microglia in regulation of ethanol neurotoxic action. Int. Rev. Neurobiol. 118, 81–103. 10.1016/B978-0-12-801284-0.00004-X 25175862

[B24] Chen J. Zhang X. Kusumo H. Costa L. G. Guizzetti M. (2013). Cholesterol efflux is differentially regulated in neurons and astrocytes: implications for brain cholesterol homeostasis. Biochim. Biophys. Acta 1831, 263–275. 10.1016/j.bbalip.2012.09.007 23010475 PMC3534809

[B25] Chinn G. A. Hirokawa K. E. Chuang T. M. Urbina C. Patel F. Fong J.. (2015). Agenesis of the corpus callosum due to defective glial wedge formation in Lhx2 mutant mice. Cereb. Cortex 25, 2707–2718. 10.1093/cercor/bhu067 24781987 PMC4537429

[B26] Christopherson K. S. Ullian E. M. Stokes C. C. Mullowney C. E. Hell J. W. Agah A.. (2005). Thrombospondins are astrocyte-secreted proteins that promote CNS synaptogenesis. Cell 120, 421–433. 10.1016/j.cell.2004.12.020 15707899

[B27] Cole G. J. Zhang C. Ojiaku P. Bell V. Devkota S. Mukhopadhyay S. (2012). Effects of ethanol exposure on nervous system development in zebrafish. Int. Rev. Cell Mol. Biol. 299, 255–315. 10.1016/B978-0-12-394310-1.00007-2 22959306

[B28] Coles C. D. Platzman K. A. Lynch M. E. Freides D. (2002). Auditory and visual sustained attention in adolescents prenatally exposed to alcohol. Alcohol. Clin. Exp. Res. 26, 263–271. 10.1111/j.1530-0277.2002.tb02533.x 11964567

[B29] Coutts D. J. Harrison N. L. (2015). Acetaldehyde, not ethanol, impairs myelin formation and viability in primary mouse oligodendrocytes. Alcohol. Clin. Exp. Res. 39, 455–462. 10.1111/acer.12642 25703384

[B30] Creeley C. E. Dikranian K. T. Johnson S. A. Farber N. B. Olney J. W. (2013). Alcohol-induced apoptosis of oligodendrocytes in the fetal macaque brain. Acta Neuropathol. Commun. 1:23. 10.1186/2051-5960-1-23 24252271 PMC3893424

[B31] Creeley C. E. Olney J. W. (2013). Drug-induced apoptosis: mechanism by which alcohol and many other drugs can disrupt brain development. Brain Sci. 3, 1153–1181. 10.3390/brainsci3031153 24587895 PMC3938204

[B32] Crews F. T. Vetreno R. P. (2014). Neuroimmune basis of alcoholic brain damage. Int. Rev. Neurobiol. 118, 315–357. 10.1016/b978-0-12-801284-0.00010-5 25175868 PMC5765863

[B33] Cudd T. A. (2005). Animal model systems for the study of alcohol teratology. Exp. Biol. Med. (Maywood) 230, 389–393. 15956768 10.1177/15353702-0323006-06

[B34] Cui Z. J. Zhao K. B. Zhao H. J. Yu D. M. Niu Y. L. Zhang J. S.. (2010). Prenatal alcohol exposure induces long-term changes in dendritic spines and synapses in the mouse visual cortex. Alcohol Alcohol. 45, 312–319. 10.1093/alcalc/agq036 20543181

[B35] Cunningham C. L. Martínez-Cerdeño V. Noctor S. C. (2013). Microglia regulate the number of neural precursor cells in the developing cerebral cortex. J. Neurosci. 33, 4216–4233. 10.1523/JNEUROSCI.3441-12.2013 23467340 PMC3711552

[B36] Dalitz P. Cock M. Harding R. Rees S. (2008). Injurious effects of acute ethanol exposure during late gestation on developing white matter in fetal sheep. Int. J. Dev. Neurosci. 26, 391–399. 10.1016/j.ijdevneu.2008.03.008 18455353

[B37] Davies D. L. Smith D. E. (1981). A Golgi study of mouse hippocampal CA1 pyramidal neurons following perinatal ethanol exposure. Neurosci. Lett. 26, 49–54. 10.1016/0304-3940(81)90424-9 7290537

[B38] Deng J. Elberger A. J. (2003). Corpus callosum and visual cortex of mice with deletion of the NMDA-NR1 receptor. II. Attenuation of prenatal alcohol exposure effects. Brain Res. Dev. Brain Res. 144, 135–150. 10.1016/s0165-3806(03)00157-3 12935911

[B39] Diedrich J. F. Minnigan H. Carp R. I. Whitaker J. N. Race R. Frey W.. (1991). Neuropathological changes in scrapie and Alzheimer’s disease are associated with increased expression of apolipoprotein E and cathepsin D in astrocytes. J. Virol. 65, 4759–4768. 1870200 10.1128/jvi.65.9.4759-4768.1991 PMC248933

[B40] Dietschy J. M. (2009). Central nervous system: cholesterol turnover, brain development and neurodegeneration. Biol. Chem. 390, 287–293. 10.1515/BC.2009.035 19166320 PMC3066069

[B41] Drew P. D. Johnson J. W. Douglas J. C. Phelan K. D. Kane C. J. (2015). Pioglitazone blocks ethanol induction of microglial activation and immune responses in the hippocampus, cerebellum and cerebral cortex in a mouse model of fetal alcohol spectrum disorders. Alcohol. Clin. Exp. Res. 39, 445–454. 10.1111/acer.12639 25703036 PMC4348240

[B42] Drew P. D. Kane C. J. (2013). “Neuroimmune mechanisms of glia and their interplay with alcohol exposure across the lifespan,” in Neural-Immune Interactions in Brain Function 359 and Alcohol Related Disorders, eds Cui C. Grandison L. Noronha A. (New York, NY: Springer), 359–386.

[B43] Drew P. D. Kane C. J. (2014). Fetal alcohol spectrum disorders and neuroimmune changes. Int. Rev. Neurobiol. 118, 41–80. 10.1016/B978-0-12-801284-0.00003-8 25175861 PMC4387770

[B44] Druse M. J. Hofteig J. H. (1977). The effect of chronic maternal alcohol consumption on the development of central nervous system myelin subfractions in rat offspring. Drug Alcohol Depend. 2, 421–429. 10.1016/0376-8716(77)90043-6 562252

[B45] du Plessis L. Jacobson S. W. Molteno C. D. Robertson F. C. Peterson B. S. Jacobson J. L.. (2014). Neural correlates of cerebellar-mediated timing during finger tapping in children with fetal alcohol spectrum disorders. Neuroimage Clin. 7, 562–570. 10.1016/j.nicl.2014.12.016 25844307 PMC4377649

[B46] El Waly B. Macchi M. Cayre M. Durbec P. (2014). Oligodendrogenesis in the normal and pathological central nervous system. Front. Neurosci. 8:145. 10.3389/fnins.2014.00145 24971048 PMC4054666

[B47] Engelhardt B. Liebner S. (2014). Novel insights into the development and maintenance of the blood-brain barrier. Cell Tissue Res. 355, 687–699. 10.1007/s00441-014-1811-2 24590145 PMC3972432

[B48] Eriksen J. L. Gillespie R. A. Druse M. J. (2000). Effects of *in utero* ethanol exposure and maternal treatment with a 5-HT(1A) agonist on S100B-containing glial cells. Brain Res. Dev. Brain Res. 121, 133–143. 10.1016/s0165-3806(00)00029-8 10876026

[B49] Fabregues I. Ferrer I. Gairi J. M. Cahuana A. Giner P. (1985). Effects of prenatal exposure to ethanol on the maturation of the pyramidal neurons in the cerebral cortex of the guinea-pig: a quantitative Golgi study. Neuropathol. Appl. Neurobiol. 11, 291–298. 10.1111/j.1365-2990.1985.tb00026.x 4058673

[B50] Fernandez-Lizarbe S. Pascual M. Guerri C. (2009). Critical role of TLR4 response in the activation of microglia induced by ethanol. J. Immunol. 183, 4733–4744. 10.4049/jimmunol.0803590 19752239

[B51] Fields R. D. (2010). Neuroscience. Change in the brain’s white matter. Science 330, 768–769. 10.1126/science.1199139 21051624 PMC3201847

[B52] Fletcher T. L. Shain W. (1993). Ethanol-induced changes in astrocyte gene expression during rat central nervous system development. Alcohol. Clin. Exp. Res. 17, 993–1001. 10.1111/j.1530-0277.1993.tb05654.x 8279687

[B53] Fryer S. L. McGee C. L. Matt G. E. Riley E. P. Mattson S. N. (2007). Evaluation of psychopathological conditions in children with heavy prenatal alcohol exposure. Pediatrics 119, e733–e741. 10.1542/peds.2006-1606 17332190

[B54] Garcia O. Torres M. Helguera P. Coskun P. Busciglio J. (2010). A role for thrombospondin-1 deficits in astrocyte-mediated spine and synaptic pathology in Down’s syndrome. PLoS One 5:e14200. 10.1371/journal.pone.0014200 21152035 PMC2996288

[B55] Giordano G. Guizzetti M. Dao K. Mattison H. A. Costa L. G. (2011). Ethanol impairs muscarinic receptor-induced neuritogenesis in rat hippocampal slices: role of astrocytes and extracellular matrix proteins. Biochem. Pharmacol. 82, 1792–1799. 10.1016/j.bcp.2011.08.014 21884684 PMC3205233

[B56] Giordano G. Pizzurro D. VanDemark K. Guizzetti M. Costa L. G. (2009). Manganese inhibits the ability of astrocytes to promote neuronal differentiation. Toxicol. Appl. Pharmacol. 240, 226–235. 10.1016/j.taap.2009.06.004 19524604

[B57] Gnaedinger J. M. Noronha A. B. Druse M. J. (1984). Myelin gangliosides in developing rats: the influence of maternal ethanol consumption. J. Neurochem. 42, 1281–1285. 10.1111/j.1471-4159.1984.tb02784.x 6707632

[B58] Goodlett C. R. Leo J. T. O’Callaghan J. P. Mahoney J. C. West J. R. (1993). Transient cortical astrogliosis induced by alcohol exposure during the neonatal brain growth spurt in rats. Brain Res. Dev. Brain Res. 72, 85–97. 10.1016/0165-3806(93)90162-4 8453767

[B59] Goodlett C. R. Peterson S. D. (1995). Sex differences in vulnerability to developmental spatial learning deficits induced by limited binge alcohol exposure in neonatal rats. Neurobiol. Learn. Mem. 64, 265–275. 10.1006/nlme.1995.0009 8564380

[B60] Goodlett C. R. Peterson S. D. Lundahl K. R. Pearlman A. D. (1997). Binge-like alcohol exposure of neonatal rats via intragastric intubation induces both Purkinje cell loss and cortical astrogliosis. Alcohol. Clin. Exp. Res. 21, 1010–1017. 10.1111/j.1530-0277.1997.tb04246.x 9309310

[B61] Granato A. Di Rocco F. Zumbo A. Toesca A. Giannetti S. (2003). Organization of cortico-cortical associative projections in rats exposed to ethanol during early postnatal life. Brain Res. Bull. 60, 339–344. 10.1016/s0361-9230(03)00052-2 12781322

[B62] Guerri C. (1998). Neuroanatomical and neurophysiological mechanisms involved in central nervous system dysfunctions induced by prenatal alcohol exposure. Alcohol. Clin. Exp. Res. 22, 304–312. 10.1111/j.1530-0277.1998.tb03653.x 9581633

[B63] Guerri C. Bazinet A. Riley E. P. (2009). Foetal alcohol spectrum disorders and alterations in brain and behaviour. Alcohol Alcohol. 44, 108–114. 10.1093/alcalc/agn105 19147799 PMC2724862

[B64] Guerri C. Pascual M. Renau-Piqueras J. (2001). Glia and fetal alcohol syndrome. Neurotoxicology 22, 593–599. 10.1016/s0161-813x(01)00037-7 11770880

[B65] Guizzetti M. Bordi F. Dieguez-Acuña F. J. Vitalone A. Madia F. Woods J. S.. (2003). Nuclear factor kappaB activation by muscarinic receptors in astroglial cells: effect of ethanol. Neuroscience 120, 941–950. 10.1016/s0306-4522(03)00401-9 12927200

[B66] Guizzetti M. Chen J. Costa L. G. (2011). “Disruption of cholesterol homeostasis in developmental neurotoxicity,” in Reproductive and Developmental Toxicology, ed. Gupta R. C. (London: Academic Press), 855–862.

[B67] Guizzetti M. Chen J. Oram J. F. Tsuji R. Dao K. Möller T.. (2007). Ethanol induces cholesterol efflux and up-regulates ATP-binding cassette cholesterol transporters in fetal astrocytes. J. Biol. Chem. 282, 18740–18749. 10.1074/jbc.m702398200 17478430

[B68] Guizzetti M. Costa L. G. (1996). Inhibition of muscarinic receptor-stimulated glial cell proliferation by ethanol. J. Neurochem. 67, 2236–2245. 10.1046/j.1471-4159.1996.67062236.x 8931454

[B69] Guizzetti M. Costa L. G. (2000). Possible role of protein kinase C zeta in muscarinic receptor-induced proliferation of astrocytoma cells. Biochem. Pharmacol. 60, 1457–1466. 10.1016/s0006-2952(00)00468-8 11020447

[B70] Guizzetti M. Costa L. G. (2002). Effect of ethanol on protein kinase Czeta and p70S6 kinase activation by carbachol: a possible mechanism for ethanol-induced inhibition of glial cell proliferation. J. Neurochem. 82, 38–46. 10.1046/j.1471-4159.2002.00942.x 12091463

[B71] Guizzetti M. Costa L. G. (2007). Cholesterol homeostasis in the developing brain: a possible new target for ethanol. Hum. Exp. Toxicol. 26, 355–360. 10.1177/0960327107078412 17615117

[B72] Guizzetti M. Moore N. H. Giordano G. Costa L. G. (2008). Modulation of neuritogenesis by astrocyte muscarinic receptors. J. Biol. Chem. 283, 31884–31897. 10.1074/jbc.M801316200 18755690 PMC2581542

[B73] Guizzetti M. Moore N. H. Giordano G. VanDeMark K. L. Costa L. G. (2010). Ethanol inhibits neuritogenesis induced by astrocyte muscarinic receptors. Glia 58, 1395–1406. 10.1002/glia.21015 20648635 PMC2925144

[B74] Guizzetti M. Thompson B. D. Kim Y. VanDeMark K. Costa L. G. (2004). Role of phospholipase D signaling in ethanol-induced inhibition of carbachol-stimulated DNA synthesis of 1321N1 astrocytoma cells. J. Neurochem. 90, 646–653. 10.1111/j.1471-4159.2004.02541.x 15255942

[B75] Guizzetti M. Zhang X. Goeke C. Gavin D. P. (2014). Glia and neurodevelopment: focus on fetal alcohol spectrum disorders. Front. Pediatr. 2:123. 10.3389/fped.2014.00123 25426477 PMC4227495

[B76] Hamilton G. F. Whitcher L. T. Klintsova A. Y. (2010). Postnatal binge-like alcohol exposure decreases dendritic complexity while increasing the density of mature spines in mPFC Layer II/III pyramidal neurons. Synapse 64, 127–135. 10.1002/syn.20711 19771589 PMC2789893

[B77] Hellemans K. G. Verma P. Yoon E. Yu W. K. Young A. H. Weinberg J. (2010). Prenatal alcohol exposure and chronic mild stress differentially alter depressive- and anxiety-like behaviors in male and female offspring. Alcohol. Clin. Exp. Res. 34, 633–645. 10.1111/j.1530-0277.2009.01132.x 20102562 PMC4833468

[B78] Higgins D. Burack M. Lein P. Banker G. (1997). Mechanisms of neuronal polarity. Curr. Opin. Neurobiol. 7, 599–604. 10.1016/S0959-4388(97)80078-5 9384542

[B79] Hirsch-Reinshagen V. Zhou S. Burgess B. L. Bernier L. McIsaac S. A. Chan J. Y.. (2004). Deficiency of ABCA1 impairs apolipoprotein E metabolism in brain. J. Biol. Chem. 279, 41197–41207. 10.1074/jbc.m407962200 15269218

[B80] Hofteig J. H. Druse M. J. (1978). Central nervous system myelination in rats exposed to ethanol *in utero*. Drug Alcohol Depend. 3, 429–434. 10.1016/0376-8716(78)90015-7 569044

[B81] Howell K. K. Lynch M. E. Platzman K. A. Smith G. H. Coles C. D. (2006). Prenatal alcohol exposure and ability, academic achievement and school functioning in adolescence: a longitudinal follow-up. J. Pediatr. Psychol. 31, 116–126. 10.1093/jpepsy/jsj029 15829611

[B82] Hoyme H. E. May P. A. Kalberg W. O. Kodituwakku P. Gossage J. P. Trujillo P. M.. (2005). A practical clinical approach to diagnosis of fetal alcohol spectrum disorders: clarification of the 1996 institute of medicine criteria. Pediatrics 115, 39–47. 10.1542/peds.2005-0702 15629980 PMC1380311

[B83] Ikonomidou C. Bittigau P. Ishimaru M. J. Wozniak D. F. Koch C. Genz K.. (2000). Ethanol-induced apoptotic neurodegeneration and fetal alcohol syndrome. Science 287, 1056–1060. 10.1126/science.287.5455.1056 10669420

[B84] Infante M. A. Moore E. M. Bischoff-Grethe A. Migliorini R. Mattson S. N. Riley E. P. (2015). Atypical cortical gyrification in adolescents with histories of heavy prenatal alcohol exposure. Brain Res. 1624, 446–454. 10.1016/j.brainres.2015.08.002 26275919 PMC4630133

[B85] Jacobs S. Doering L. C. (2010). Astrocytes prevent abnormal neuronal development in the fragile x mouse. J. Neurosci. 30, 4508–4514. 10.1523/JNEUROSCI.5027-09.2010 20335488 PMC6634485

[B86] Jones K. L. Smith D. W. (1973). Recognition of the fetal alcohol syndrome in early infancy. Lancet 302, 999–1001. 10.1016/s0140-6736(73)91092-1 4127281

[B87] Jones K. L. Smith D. W. (1975). The fetal alcohol syndrome. Teratology 12, 1–10. 10.1002/tera.1420120102 1162620

[B88] Jones K. L. Smith D. W. Ulleland C. N. Streissguth P. (1973). Pattern of malformation in offspring of chronic alcoholic mothers. Lancet 1, 1267–1271. 10.1016/s0140-6736(73)91291-9 4126070

[B89] Joseph J. Warton C. Jacobson S. W. Jacobson J. L. Molteno C. D. Eicher A.. (2014). Three-dimensional surface deformation-based shape analysis of hippocampus and caudate nucleus in children with fetal alcohol spectrum disorders. Hum. Brain Mapp. 35, 659–672. 10.1002/hbm.22209 23124690 PMC6869198

[B90] Kane C. J. Phelan K. D. Han L. Smith R. R. Xie J. Douglas J. C.. (2011). Protection of neurons and microglia against ethanol in a mouse model of fetal alcohol spectrum disorders by peroxisome proliferator-activated receptor-gamma agonists. Brain Behav. Immun. 25, S137–S145. 10.1016/j.bbi.2011.02.016 21376806 PMC3104506

[B91] Kelly S. J. Goodlett C. R. Hannigan J. H. (2009). Animal models of fetal alcohol spectrum disorders: impact of the social environment. Dev. Disabil. Res. Rev. 15, 200–208. 10.1002/ddrr.69 19731387 PMC3598020

[B92] Kelly S. J. Goodlett C. R. Hulsether S. A. West J. R. (1988). Impaired spatial navigation in adult female but not adult male rats exposed to alcohol during the brain growth spurt. Behav. Brain Res. 27, 247–257. 10.1016/0166-4328(88)90121-0 3358862

[B93] Kim W. S. Rahmanto A. S. Kamili A. Rye K. A. Guillemin G. J. Gelissen I. C.. (2007). Role of ABCG1 and ABCA1 in regulation of neuronal cholesterol efflux to apolipoprotein E discs and suppression of amyloid-beta peptide generation. J. Biol. Chem. 282, 2851–2861. 10.1074/jbc.m607831200 17121837

[B94] Koldamova R. P. Lefterov I. M. Ikonomovic M. D. Skoko J. Lefterov P. I. Isanski B. A.. (2003). 22R-hydroxycholesterol and 9-cis-retinoic acid induce ATP-binding cassette transporter A1 expression and cholesterol efflux in brain cells and decrease amyloid beta secretion. J. Biol. Chem. 278, 13244–13256. 10.1074/jbc.m300044200 12547833

[B95] Kötter K. Klein J. (1999). Ethanol inhibits astroglial cell proliferation by disruption of phospholipase D-mediated signaling. J. Neurochem. 73, 2517–2523. 10.1046/j.1471-4159.1999.0732517.x 10582613

[B96] Lancaster F. E. (1994). Alcohol and white matter development–a review. Alcohol. Clin. Exp. Res. 18, 644–647. 10.1111/j.1530-0277.1994.tb00924.x 7943669

[B97] Lebel C. Roussotte F. Sowell E. R. (2011). Imaging the impact of prenatal alcohol exposure on the structure of the developing human brain. Neuropsychol. Rev. 21, 102–118. 10.1007/s11065-011-9163-0 21369875 PMC3098972

[B98] Lebel C. Walker L. Leemans A. Phillips L. Beaulieu C. (2008). Microstructural maturation of the human brain from childhood to adulthood. Neuroimage 40, 1044–1055. 10.1016/j.neuroimage.2007.12.053 18295509

[B99] Liu M. Oyarzabal E. A. Yang R. Murphy S. J. Hurn P. D. (2008). A novel method for assessing sex-specific and genotype-specific response to injury in astrocyte culture. J. Neurosci. Methods 171, 214–217. 10.1016/j.jneumeth.2008.03.002 18436308 PMC2701697

[B100] Livy D. J. Elberger A. J. (2008). Alcohol exposure during the first two trimesters-equivalent alters the development of corpus callosum projection neurons in the rat. Alcohol 42, 285–293. 10.1016/j.alcohol.2008.04.002 18468834 PMC2683683

[B101] Lokhorst D. K. Druse M. J. (1993). Effects of ethanol on cultured fetal serotonergic neurons. Alcohol. Clin. Exp. Res. 17, 86–93. 10.1111/j.1530-0277.1993.tb00730.x 8383927

[B102] Lövblad K. Ramelli G. Remonda L. Nirkko A. C. Ozdoba C. Schroth G. (1997). Retardation of myelination due to dietary vitamin B12 deficiency: cranial MRI findings. Pediatr. Radiol. 27, 155–158. 10.1007/s002470050090 9028851

[B103] Luo J. (2012). Mechanisms of ethanol-induced death of cerebellar granule cells. Cerebellum 11, 145–154. 10.1007/s12311-010-0219-0 20927663 PMC3355343

[B104] Martín M. G. Pfrieger F. Dotti C. G. (2014). Cholesterol in brain disease: sometimes determinant and frequently implicated. EMBO Rep. 15, 1036–1052. 10.15252/embr.201439225 25223281 PMC4253844

[B105] Marín-Teva J. L. Cuadros M. A. Martín-Oliva D. Navascués J. (2011). Microglia and neuronal cell death. Neuron Glia Biol. 7, 25–40. 10.1017/S1740925X12000014 22377033

[B106] Martinez R. Gomes F. C. (2002). Neuritogenesis induced by thyroid hormone-treated astrocytes is mediated by epidermal growth factor/mitogen-activated protein kinase-phosphatidylinositol 3-kinase pathways and involves modulation of extracellular matrix proteins. J. Biol. Chem. 277, 49311–49318. 10.1074/jbc.m209284200 12356760

[B107] Mattson S. N. Crocker N. Nguyen T. T. (2011). Fetal alcohol spectrum disorders: neuropsychological and behavioral features. Neuropsychol. Rev. 21, 81–101. 10.1007/s11065-011-9167-9 21503685 PMC3410672

[B108] Mattson S. N. Riley E. P. (1998). A review of the neurobehavioral deficits in children with fetal alcohol syndrome or prenatal exposure to alcohol. Behav. Brain Res. 22, 279–294. 10.1111/j.1530-0277.1998.tb03651.x 9581631

[B109] Mattson S. N. Riley E. P. Jernigan T. L. Ehlers C. L. Delis D. C. Jones K. L.. (1992). Fetal alcohol syndrome: a case report of neuropsychological, MRI and EEG assessment of two children. Alcohol. Clin. Exp. Res. 16, 1001–1003. 10.1111/j.1530-0277.1992.tb01909.x 1443415

[B110] May P. A. Gossage J. P. Kalberg W. O. Robinson L. K. Buckley D. Manning M.. (2009). Prevalence and epidemiologic characteristics of FASD from various research methods with an emphasis on recent in-school studies. Dev. Disabil. Res. Rev. 15, 176–192. 10.1002/ddrr.68 19731384

[B111] May P. A. Gossage J. P. Marais A. S. Adnams C. M. Hoyme H. E. Jones K. L.. (2007). The epidemiology of fetal alcohol syndrome and partial FAS in a South African community. Drug Alcohol Depend. 88, 259–271. 10.1016/j.drugalcdep.2006.11.007 17127017 PMC1865526

[B112] McClure K. D. French R. L. Heberlein U. (2011). A Drosophila model for fetal alcohol syndrome disorders: role for the insulin pathway. Dis. Model. Mech. 4, 335–346. 10.1242/dmm.006411 21303840 PMC3097455

[B113] Medina A. E. (2011). Fetal alcohol spectrum disorders and abnormal neuronal plasticity. Neuroscientist 17, 274–287. 10.1177/1073858410383336 21383101 PMC3337631

[B114] Melo P. Pinazo-Durán M. D. Salgado-Borges J. Tavares M. A. (2008). Correlation of axon size and myelin occupancy in rats prenatally exposed to methamphetamine. Brain Res. 1222, 61–68. 10.1016/j.brainres.2008.05.047 18585694

[B115] Messing A. Brenner M. Feany M. B. Nedergaard M. Goldman J. E. (2012). Alexander disease. J. Neurosci. 32, 5017–5023. 10.1523/JNEUROSCI.5384-11.2012 22496548 PMC3336214

[B116] Meyer U. (2013). Developmental neuroinflammation and schizophrenia. Prog. Neuropsychopharmacol. Biol. Psychiatry 42, 20–34. 10.1016/j.pnpbp.2011.11.003 22122877

[B117] Migliorini R. Moore E. M. Glass L. Infante M. A. Tapert S. F. Jones K. L.. (2015). Anterior cingulate cortex surface area relates to behavioral inhibition in adolescents with and without heavy prenatal alcohol exposure. Behav. Brain Res. 292, 26–35. 10.1016/j.bbr.2015.05.037 26025509 PMC4558293

[B118] Miller M. W. (1986). Effects of alcohol on the generation and migration of cerebral cortical neurons. Science 233, 1308–1311. 10.1126/science.3749878 3749878

[B119] Miller M. W. (2007). Exposure to ethanol during gastrulation alters somatosensory-motor cortices and the underlying white matter in the macaque. Cereb. Cortex 17, 2961–2971. 10.1093/cercor/bhm024 17389626

[B120] Miller M. W. Potempa G. (1990). Numbers of neurons and glia in mature rat somatosensory cortex: effects of prenatal exposure to ethanol. J. Comp. Neurol. 293, 92–102. 10.1002/cne.902930108 2312794

[B121] Miller M. W. Robertson S. (1993). Prenatal exposure to ethanol alters the postnatal development and transformation of radial glia to astrocytes in the cortex. J. Comp. Neurol. 337, 253–266. 10.1002/cne.903370206 8276999

[B122] Mitew S. Hay C. M. Peckham H. Xiao J. Koenning M. Emery B. (2013). Mechanisms regulating the development of oligodendrocytes and central nervous system myelin. Neuroscience 276, 29–47. 10.1016/j.neuroscience.2013.11.029 24275321

[B123] Montoliu C. Sancho-Tello M. Azorin I. Burgal M. Vallés S. Renau-Piqueras J.. (1995). Ethanol increases cytochrome P4502E1 and induces oxidative stress in astrocytes. J. Neurochem. 65, 2561–2570. 10.1046/j.1471-4159.1995.65062561.x 7595552

[B124] Mooney S. M. Miller M. W. (2007). Nerve growth factor neuroprotection of ethanol-induced neuronal death in rat cerebral cortex is age dependent. Neuroscience 149, 372–381. 10.1016/j.neuroscience.2007.08.012 17869443 PMC2128252

[B125] Moore N. H. Costa L. G. Shaffer S. A. Goodlett D. R. Guizzetti M. (2009). Shotgun proteomics implicates extracellular matrix proteins and protease systems in neuronal development induced by astrocyte cholinergic stimulation. J. Neurochem. 108, 891–908. 10.1111/j.1471-4159.2008.05836.x 19077055 PMC2754805

[B126] Morgan J. T. Chana G. Pardo C. A. Achim C. Semendeferi K. Buckwalter J.. (2010). Microglial activation and increased microglial density observed in the dorsolateral prefrontal cortex in autism. Biol. Psychiatry 68, 368–376. 10.1016/j.biopsych.2010.05.024 20674603

[B127] Nadarajah B. Parnavelas J. G. (2002). Modes of neuronal migration in the developing cerebral cortex. Nat. Rev. Neurosci. 3, 423–432. 10.1038/nrn845 12042877

[B128] Nakagawa Y. Chiba K. (2015). Diversity and plasticity of microglial cells in psychiatric and neurological disorders. Pharmacol. Ther. 154, 21–35. 10.1016/j.pharmthera.2015.06.010 26129625

[B129] Nakai M. Kawamata T. Taniguchi T. Maeda K. Tanaka C. (1996). Expression of apolipoprotein E mRNA in rat microglia. Neurosci. Lett. 211, 41–44. 10.1016/0304-3940(96)12716-6 8809843

[B130] Nash R. Krishnamoorthy M. Jenkins A. Csete M. (2012). Human embryonic stem cell model of ethanol-mediated early developmental toxicity. Exp. Neurol. 234, 127–135. 10.1016/j.expneurol.2011.12.022 22227564

[B131] Nave K. A. (2010). Myelination and support of axonal integrity by glia. Nature 468, 244–252. 10.1038/nature09614 21068833

[B132] Norman A. L. Crocker N. Mattson S. N. Riley E. P. (2009). Neuroimaging and fetal alcohol spectrum disorders. Dev. Disabil. Res. Rev. 15, 209–217. 10.1002/ddrr.72 19731391 PMC3442778

[B133] Norris C. R. Kalil K. (1991). Guidance of callosal axons by radial glia in the developing cerebral cortex. J. Neurosci. 11, 3481–3492. 1941093 10.1523/JNEUROSCI.11-11-03481.1991 PMC6575559

[B134] Nowaczyk M. J. Whelan D. T. Heshka T. W. Hill R. E. (1999). Smith-Lemli-Opitz syndrome: a treatable inherited error of metabolism causing mental retardation. CMAJ 161, 165–170. 10439827 PMC1230468

[B135] Nualart-Marti A. Solsona C. Fields R. D. (2013). Gap junction communication in myelinating glia. Biochim. Biophys. Acta 1828, 69–78. 10.1016/j.bbamem.2012.01.024 22326946 PMC4474145

[B136] Obermeier B. Daneman R. Ransohoff R. M. (2013). Development, maintenance and disruption of the blood-brain barrier. Nat. Med. 19, 1584–1596. 10.1038/nm.3407 24309662 PMC4080800

[B137] Paolicelli R. C. Bolasco G. Pagani F. Maggi L. Scianni M. Panzanelli P.. (2011). Synaptic pruning by microglia is necessary for normal brain development. Science 333, 1456–1458. 10.1126/science.1202529 21778362

[B138] Paolozza A. Munn R. Munoz D. P. Reynolds J. N. (2015). Eye movements reveal sexually dimorphic deficits in children with fetal alcohol spectrum disorder. Front. Neurosci. 9:76. 10.3389/fnins.2015.00076 25814922 PMC4356081

[B139] Parson S. H. Dhillon B. Findlater G. S. Kaufman M. H. (1995). Optic nerve hypoplasia in the fetal alcohol syndrome: a mouse model. J. Anat. 186, 313–320. 7649829 PMC1167188

[B140] Pascual O. Casper K. B. Kubera C. Zhang J. Revilla-Sanchez R. Sul J. Y.. (2005). Astrocytic purinergic signaling coordinates synaptic networks. Science 310, 113–116. 10.1126/science.1116916 16210541

[B141] Pascual M. Guerri C. (2007). The peptide NAP promotes neuronal growth and differentiation through extracellular signal-regulated protein kinase and Akt pathways and protects neurons co-cultured with astrocytes damaged by ethanol. J. Neurochem. 103, 557–568. 10.1111/j.1471-4159.2007.04761.x 17623041

[B142] Patten A. R. Fontaine C. J. Christie B. R. (2014). A comparison of the different animal models of fetal alcohol spectrum disorders and their use in studying complex behaviors. Front. Pediatr. 2:93. 10.3389/fped.2014.00093 25232537 PMC4153370

[B143] Paul A. P. Medina A. E. (2012). Overexpression of serum response factor in astrocytes improves neuronal plasticity in a model of early alcohol exposure. Neuroscience 221, 193–202. 10.1016/j.neuroscience.2012.06.045 22742904 PMC3504719

[B144] Perry V. H. Teeling J. (2013). Microglia and macrophages of the central nervous system: the contribution of microglia priming and systemic inflammation to chronic neurodegeneration. Semin. Immunopathol. 35, 601–612. 10.1007/s00281-013-0382-8 23732506 PMC3742955

[B145] Pfrieger F. W. Barres B. A. (1997). Synaptic efficacy enhanced by glial cells *in vitro*. Science 277, 1684–1687. 10.1126/science.277.5332.1684 9287225

[B146] Phillips D. E. (1994). “Research monograph no. 27. Effects of alcohol on glial development *in vivo*: morphological studies,” in Alcohol and Glial Cells, ed. Lancaster F. E. (Bethesda, MD: National Institute of Health, NIAAA), 69–91.

[B147] Phillips D. E. Krueger S. K. (1992). Effects of combined pre- and postnatal ethanol exposure (three trimester equivalency) on glial cell development in rat optic nerve. Int. J. Dev. Neurosci. 10, 197–206. 10.1016/0736-5748(92)90059-9 1442168

[B148] Pinazo-Duran M. D. Renau-Piqueras J. Guerri C. Strömland K. (1997). Optic nerve hypoplasia in fetal alcohol syndrome: an update. Eur. J. Ophthalmol. 7, 262–270. 9352281 10.1177/112067219700700311

[B149] Pizzurro D. M. Dao K. Costa L. G. (2014). Diazinon and diazoxon impair the ability of astrocytes to foster neurite outgrowth in primary hippocampal neurons. Toxicol. Appl. Pharmacol. 274, 372–382. 10.1016/j.taap.2013.11.023 24342266 PMC3916905

[B150] Qiang M. Wang M. W. Elberger A. J. (2002). Second trimester prenatal alcohol exposure alters development of rat corpus callosum. Neurotoxicol. Teratol. 24, 719–732. 10.1016/s0892-0362(02)00267-2 12460654

[B151] Quinlan R. A. Brenner M. Goldman J. E. Messing A. (2007). GFAP and its role in Alexander disease. Exp. Cell Res. 313, 2077–2087. 10.1016/j.yexcr.2007.04.004 17498694 PMC2702672

[B152] Rakic P. (1972). Mode of cell migration to the superficial layers of fetal monkey neocortex. J. Comp. Neurol. 145, 61–83. 10.1002/cne.901450105 4624784

[B153] Rasmussen C. Benz J. Pei J. Andrew G. Schuller G. Abele-Webster L.. (2010). The impact of an ADHD co-morbidity on the diagnosis of FASD. Can. J. Clin. Pharmacol. 17, e165–e176. 20395649

[B154] Rathinam M. L. Watts L. T. Stark A. A. Mahimainathan L. Stewart J. Schenker S.. (2006). Astrocyte control of fetal cortical neuron glutathione homeostasis: up-regulation by ethanol. J. Neurochem. 96, 1289–1300. 10.1111/j.1471-4159.2006.03674.x 16464233

[B155] Renau-Piqueras J. Zaragoza R. De Paz P. Baguena-Cervellera R. Megias L. Guerri C. (1989). Effects of prolonged ethanol exposure on the glial fibrillary acidic protein-containing intermediate filaments of astrocytes in primary culture: a quantitative immunofluorescence and immunogold electron microscopic study. J. Histochem. Cytochem. 37, 229–240. 10.1177/37.2.2642942 2642942

[B156] Resnicoff M. Rubini M. Baserga R. Rubin R. (1994). Ethanol inhibits insulin-like growth factor-1-mediated signalling and proliferation of C6 rat glioblastoma cells. Lab. Invest. 71, 657–662. 7526037

[B157] Rice D. Barone S. Jr. (2000). Critical periods of vulnerability for the developing nervous system: evidence from humans and animal models. Environ. Health Perspect. 108(Suppl. 3), 511–533. 10.2307/3454543 10852851 PMC1637807

[B158] Riley E. P. Mattson S. N. Sowell E. R. Jernigan T. L. Sobel D. F. Jones K. L. (1995). Abnormalities of the corpus callosum in children prenatally exposed to alcohol. Alcohol. Clin. Exp. Res. 19, 1198–1202. 10.1111/j.1530-0277.1995.tb01600.x 8561290

[B159] Riley E. P. McGee C. L. (2005). Fetal alcohol spectrum disorders: an overview with emphasis on changes in brain and behavior. Exp. Biol. Med. (Maywood) 230, 357–365. 15956765 10.1177/15353702-0323006-03

[B160] Rodriguez J. I. Kern J. K. (2011). Evidence of microglial activation in autism and its possible role in brain underconnectivity. Neuron Glia Biol. 7, 205–213. 10.1017/s1740925X12000142 22874006 PMC3523548

[B161] Rosenbluth J. (1999). A brief history of myelinated nerve fibers: one hundred and fifty years of controversy. J. Neurocytol. 28, 251–262. 10.1023/A:1007083409850 10739568

[B162] Rowitch D. H. Kriegstein A. R. (2010). Developmental genetics of vertebrate glial-cell specification. Nature 468, 214–222. 10.1038/nature09611 21068830

[B163] Rubert G. Miñana R. Pascual M. Guerri C. (2006). Ethanol exposure during embryogenesis decreases the radial glial progenitorpool and affects the generation of neurons and astrocytes. J. Neurosci. Res. 84, 483–496. 10.1002/jnr.20963 16770775

[B164] Ryabinin A. E. Cole M. Bloom F. E. Wilson M. C. (1995). Exposure of neonatal rats to alcohol by vapor inhalation demonstrates specificity of microcephaly and Purkinje cell loss but not astrogliosis. Alcohol. Clin. Exp. Res. 19, 784–791. 10.1111/j.1530-0277.1995.tb01583.x 7573809

[B165] Saez R. Burgal M. Renau-Piqueras J. Marqués A. Guerri C. (1991). Evolution of several cytoskeletal proteins of astrocytes in primary culture: effect of prenatal alcohol exposure. Neurochem. Res. 16, 737–747. 10.1007/bf00965682 1944762

[B166] Saijo K. Glass C. K. (2011). Microglial cell origin and phenotypes in health and disease. Nat. Rev. Immunol. 11, 775–787. 10.1038/nri3086 22025055

[B167] Samorajski T. Lancaster F. Wiggins R. C. (1986). Fetal ethanol exposure: a morphometric analysis of myelination in the optic nerve. Int. J. Dev. Neurosci. 4, 369–374. 10.1016/0736-5748(86)90054-7 3455596

[B168] Sanchez E. S. Bigbee J. W. Fobbs W. Robinson S. E. Sato-Bigbee C. (2008). Opioid addiction and pregnancy: perinatal exposure to buprenorphine affects myelination in the developing brain. Glia 56, 1017–1027. 10.1002/glia.20675 18381654 PMC2577583

[B169] Santos-Galindo M. Acaz-Fonseca E. Bellini M. J. Garcia-Segura L. M. (2011). Sex differences in the inflammatory response of primary astrocytes to lipopolysaccharide. Biol. Sex Differ. 2:7. 10.1186/2042-6410-2-7 21745355 PMC3143074

[B170] Sarkar D. K. Kuhn P. Marano J. Chen C. Boyadjieva N. (2007). Alcohol exposure during the developmental period induces beta-endorphin neuronal death and causes alteration in the opioid control of stress axis function. Endocrinology 148, 2828–2834. 10.1210/en.2006-1606 17347308

[B171] Sherwood C. C. Stimpson C. D. Raghanti M. A. Wildman D. E. Uddin M. Grossman L. I.. (2006). Evolution of increased glia-neuron ratios in the human frontal cortex. Proc. Natl. Acad. Sci. U S A 103, 13606–13611. 10.1073/pnas.0605843103 16938869 PMC1564260

[B172] Shetty A. K. Phillips D. E. (1992). Effects of prenatal ethanol exposure on the development of Bergmann glia and astrocytes in the rat cerebellum: an immunohistochemical study. J. Comp. Neurol. 321, 19–32. 10.1002/cne.903210103 1613136

[B173] Shu T. Puche A. C. Richards L. J. (2003). Development of midline glial populations at the corticoseptal boundary. J. Neurobiol. 57, 81–94. 10.1002/neu.10252 12973830

[B174] Sild M. Ruthazer E. S. (2011). Radial glia: progenitor, pathway and partner. Neuroscientist 17, 288–302. 10.1177/1073858410385870 21558559

[B175] Simons M. Trotter J. (2007). Wrapping it up: the cell biology of myelination. Curr. Opin. Neurobiol. 17, 533–540. 10.1016/j.conb.2007.08.003 17923405

[B176] Sloan S. A. Barres B. A. (2014). Mechanisms of astrocyte development and their contributions to neurodevelopmental disorders. Curr. Opin. Neurobiol. 27, 75–81. 10.1016/j.conb.2014.03.005 24694749 PMC4433289

[B177] Smith S. M. (2008). The avian embryo in fetal alcohol research. Methods Mol. Biol. 447, 75–84. 10.1007/978-1-59745-242-7_6 18369912

[B178] Smith D. E. Davies D. L. (1990). Effect of perinatal administration of ethanol on the CA1 pyramidal cell of the hippocampus and Purkinje cell of the cerebellum: an ultrastructural survey. J. Neurocytol. 19, 708–717. 10.1007/bf01188039 2077112

[B179] Sofroniew M. V. (2014). Astrogliosis. Cold Spring Harb. Perspect. Biol. 7:a020420. 10.1101/cshperspect.a020420 25380660 PMC4315924

[B180] Soh D. W. Skocic J. Nash K. Stevens S. Turner G. R. Rovet J. (2015). Self-regulation therapy increases frontal gray matter in children with fetal alcohol spectrum disorder: evaluation by voxel-based morphometry. Front. Hum. Neurosci. 9:108. 10.3389/fnhum.2015.00108 25788884 PMC4349084

[B181] Sowell E. R. Johnson A. Kan E. Lu L. H. Van Horn J. D. Toga A. W.. (2008). Mapping white matter integrity and neurobehavioral correlates in children with fetal alcohol spectrum disorders. J. Neurosci. 28, 1313–1319. 10.1523/JNEUROSCI.5067-07.2008 18256251 PMC3567846

[B182] Sowell E. R. Thompson P. M. Mattson S. N. Tessner K. D. Jernigan T. L. Riley E. P.. (2001). Voxel-based morphometric analyses of the brain in children and adolescents prenatally exposed to alcohol. Neuroreport 12, 515–523. 10.1097/00001756-200103050-00018 11234756

[B183] Steinhauser C. Kettenmann H. (2009). “Astrocyte: neurotransmitter and hormone receptors,” in Reference Module in Biomedical Sciences Encyclopedia of Neuroscience, ed. Caplan M. (London: Academic Press), 579–585.

[B184] Stellwagen D. Malenka R. C. (2006). Synaptic scaling mediated by glial TNF-alpha. Nature 440, 1054–1059. 10.1038/nature04671 16547515

[B185] Stratton K. Howe C. Battaglia F. C. (1996). Fetal Alcohol Syndrome: Diagnosis, Epidemiology, Prevention and Treatment. Washington, DC: National Academy Press.

[B186] Streissguth A. P. Barr H. M. Kogan J. Bookstein F. L. (1997). “Primary and secondary disabilities,” in Fetal Alcohol Syndrome in the Challenge of Fetal Alcohol Syndrome: Overcoming Secondary Disabilities, eds Streissguth A. P. Kanter J. (Seattle, WA: University of Washington Press), 25–39.

[B187] Strömland K. (2004). Visual impairment and ocular abnormalities in children with fetal alcohol syndrome. Addict. Biol. 9, 153–157; discussion 159–160. 10.1080/13556210410001717024 15223541

[B188] Suárez R. Gobius I. Richards L. J. (2014). Evolution and development of interhemispheric connections in the vertebrate forebrain. Front. Hum. Neurosci. 8:497. 10.3389/fnhum.2014.00497 25071525 PMC4094842

[B189] Sulik K. K. (2005). Genesis of alcohol-induced craniofacial dysmorphism. Exp. Biol. Med. (Maywood) 230, 366–375. 15956766 10.1177/15353702-0323006-04

[B190] Suzuki K. Sugihara G. Ouchi Y. Nakamura K. Futatsubashi M. Takebayashi K.. (2013). Microglial activation in young adults with autism spectrum disorder. JAMA Psychiatry 70, 49–58. 10.1001/jamapsychiatry.2013.272 23404112

[B191] Szuchet S. Nielsen L. L. Domowicz M. S. Austin J. R. 2nd Arvanitis D. L. (2015). CNS myelin sheath is stochastically built by homotypic fusion of myelin membranes within the bounds of an oligodendrocyte process. J. Struct. Biol. 190, 56–72. 10.1016/j.jsb.2015.01.015 25682762

[B192] Tajuddin N. F. Druse M. J. (1999). *In utero* ethanol exposure decreased the density of serotonin neurons. Maternal ipsapirone treatment exerted a protective effect. Brain Res. Dev. Brain Res. 117, 91–97. 10.1016/s0165-3806(99)00102-9 10536236

[B193] Tajuddin N. F. Druse M. J. (2001). A persistent deficit of serotonin neurons in the offspring of ethanol-fed dams: protective effects of maternal ipsapirone treatment. Brain Res. Dev. Brain Res. 129, 181–188. 10.1016/s0165-3806(01)00199-7 11506862

[B194] Tajuddin N. F. Orrico L. A. Eriksen J. L. Druse M. J. (2003). Effects of ethanol and ipsapirone on the development of midline raphe glial cells and astrocytes. Alcohol 29, 157–164. 10.1016/s0741-8329(03)00024-7 12798971

[B195] Taléns-Visconti R. Sanchez-Vera I. Kostic J. Perez-Arago M. A. Erceg S. Stojkovic M.. (2011). Neural differentiation from human embryonic stem cells as a tool to study early brain development and the neuroteratogenic effects of ethanol. Stem Cells Dev. 20, 327–339. 10.1089/scd.2010.0037 20491543

[B196] Tomás M. Marín P. Megías L. Egea G. Renau-Piqueras J. (2005). Ethanol perturbs the secretory pathway in astrocytes. Neurobiol. Dis. 20, 773–784. 10.1016/j.nbd.2005.05.012 15953732

[B197] Topper L. A. Baculis B. C. Valenzuela C. F. (2015). Exposure of neonatal rats to alcohol has differential effects on neuroinflammation and neuronal survival in the cerebellum and hippocampus. J. Neuroinflammation 12:160. 10.1186/s12974-015-0382-9 26337952 PMC4558631

[B198] Tsuji R. Guizzetti M. Costa L. G. (2003). *In vivo* ethanol decreases phosphorylated MAPK and p70S6 kinase in the developing rat brain. Neuroreport 14, 1395–1399. 10.1097/00001756-200307180-00023 12876481

[B199] Ullian E. M. Sapperstein S. K. Christopherson K. S. Barres B. A. (2001). Control of synapse number by glia. Science 291, 657–661. 10.1126/science.291.5504.657 11158678

[B200] Vallés S. Pitarch J. Renau-Piqueras J. Guerri C. (1997). Ethanol exposure affects glial fibrillary acidic protein gene expression and transcription during rat brain development. J. Neurochem. 69, 2484–2493. 10.1046/j.1471-4159.1997.69062484.x 9375681

[B201] Valles S. Sancho-Tello M. Miñana R. Climent E. Renau-Piqueras J. Guerri C. (1996). Glial fibrillary acidic protein expression in rat brain and in radial glia culture is delayed by prenatal ethanol exposure. J. Neurochem. 67, 2425–2433. 10.1046/j.1471-4159.1996.67062425.x 8931475

[B202] Van Hartesveldt C. Moore B. Hartman B. K. (1986). Transient midline raphe glial structure in the developing rat. J. Comp. Neurol. 253, 174–184. 10.1002/cne.902530205 3540038

[B203] Varlinskaya E. I. Mooney S. M. (2014). Acute exposure to ethanol on gestational day 15 affects social motivation of female offspring. Behav. Brain Res. 261, 106–109. 10.1016/j.bbr.2013.12.016 24355753 PMC3927448

[B204] Vemuri M. C. Chetty C. S. (2005). Alcohol impairs astrogliogenesis by stem cells in rodent neurospheres. Neurochem. Int. 47, 129–135. 10.1016/j.neuint.2005.04.019 15904994

[B205] Wahrle S. E. Jiang H. Parsadanian M. Legleiter J. Han X. Fryer J. D.. (2004). ABCA1 is required for normal central nervous system ApoE levels and for lipidation of astrocyte-secreted apoE. J. Biol. Chem. 279, 40987–40993. 10.1074/jbc.m407963200 15269217

[B206] Warren K. R. Hewitt B. G. Thomas J. D. (2011). Fetal alcohol spectrum disorders: research challenges and opportunities. Alcohol Res. Health. 34, 4–14. 23580035 PMC3756137

[B207] Watts L. T. Rathinam M. L. Schenker S. Henderson G. I. (2005). Astrocytes protect neurons from ethanol-induced oxidative stress and apoptotic death. J. Neurosci. Res. 80, 655–666. 10.1002/jnr.20502 15880562

[B208] Weinberg J. Sliwowska J. H. Lan N. Hellemans K. G. (2008). Prenatal alcohol exposure: foetal programming, the hypothalamic-pituitary-adrenal axis and sex differences in outcome. J. Neuroendocrinol. 20, 470–488. 10.1111/j.1365-2826.2008.01669.x 18266938 PMC8942074

[B209] West J. R. Hamre K. M. Cassell M. D. (1986). Effects of ethanol exposure during the third trimester equivalent on neuron number in rat hippocampus and dentate gyrus. Alcohol. Clin. Exp. Res. 10, 190–197. 10.1111/j.1530-0277.1986.tb05070.x 3521377

[B210] Whitcher L. T. Klintsova A. Y. (2008). Postnatal binge-like alcohol exposure reduces spine density without affecting dendritic morphology in rat mPFC. Synapse 62, 566–573. 10.1002/syn.20532 18512209 PMC10156950

[B211] Wilhelm C. J. Hashimoto J. Roberts M. Bloom S. H. Andrew M. Wiren K. (2015). Astrocyte dysfunction induced by alcohol in females but not males. Brain Pathol. [Epub ahead of print]. 10.1111/bpa.12276 26088166 PMC8028916

[B212] Willford J. Day R. Aizenstein H. Day N. (2010). Caudate asymmetry: a neurobiological marker of moderate prenatal alcohol exposure in young adults. Neurotoxicol. Teratol. 32, 589–594. 10.1016/j.ntt.2010.06.012 20609385 PMC2975743

[B213] Xie A. X. Petravicz J. McCarthy K. D. (2015). Molecular approaches for manipulating astrocytic signaling *in vivo*. Front. Cell. Neurosci. 9:144. 10.3389/fncel.2015.00144 25941472 PMC4403552

[B214] Yang Y. Ge W. Chen Y. Zhang Z. Shen W. Wu C.. (2003). Contribution of astrocytes to hippocampal long-term potentiation through release of D-serine. Proc. Natl. Acad. Sci. U S A 100, 15194–15199. 10.1073/pnas.2431073100 14638938 PMC299953

[B215] Yanni P. A. Rising L. J. Ingraham C. A. Lindsley T. A. (2002). Astrocyte-derived factors modulate the inhibitory effect of ethanol on dendritic development. Glia 38, 292–302. 10.1002/glia.10071.abs 12007142

[B216] Young K. M. Psachoulia K. Tripathi R. B. Dunn S. J. Cossell L. Attwell D.. (2013). Oligodendrocyte dynamics in the healthy adult CNS: evidence for myelin remodeling. Neuron 77, 873–885. 10.1016/j.neuron.2013.01.006 23473318 PMC3842597

[B217] Zhang X. Bhattacharyya S. Kusumo H. Goodlett C. R. Tobacman J. K. Guizzetti M. (2014). Arylsulfatase B modulates neurite outgrowth via astrocyte chondroitin-4-sulfate: dysregulation by ethanol. Glia 62, 259–271. 10.1002/glia.22604 24311516 PMC4272875

[B218] Zhang X. Lan N. Bach P. Nordstokke D. Yu W. Ellis L.. (2012). Prenatal alcohol exposure alters the course and severity of adjuvant-induced arthritis in female rats. Brain Behav. Immun. 26, 439–450. 10.1016/j.bbi.2011.11.005 22155498 PMC3319741

[B219] Zhou C. Chen J. Zhang X. Costa L. G. Guizzetti M. (2014). Prenatal ethanol exposure up-regulates the cholesterol transporters ATP-binding cassette A1 and G1 and reduces cholesterol levels in the developing rat brain. Alcohol Alcohol. 49, 626–634. 10.1093/alcalc/agu049 25081040 PMC4834908

[B220] Zhou F. C. Sari Y. Zhang J. K. Goodlett C. R. Li T. (2001). Prenatal alcohol exposure retards the migration and development of serotonin neurons in fetal C57BL mice. Brain Res. Dev. Brain Res. 126, 147–155. 10.1016/s0165-3806(00)00144-9 11248348

[B221] Zoeller R. T. Butnariu O. V. Fletcher D. L. Riley E. P. (1994). Limited postnatal ethanol exposure permanently alters the expression of mRNAS encoding myelin basic protein and myelin-associated glycoprotein in cerebellum. Alcohol. Clin. Exp. Res. 18, 909–916. 10.1111/j.1530-0277.1994.tb00059.x 7526726

